# Incomplete lineage sorting rather than hybridization explains the inconsistent phylogeny of the wisent

**DOI:** 10.1038/s42003-018-0176-6

**Published:** 2018-10-19

**Authors:** Kun Wang, Johannes A. Lenstra, Liang Liu, Quanjun Hu, Tao Ma, Qiang Qiu, Jianquan Liu

**Affiliations:** 1 State Key Laboratory of Grassland Agro-ecosystem, Institute of Innovation Ecology & College of Life Sciences, Lanzhou University, Lanzhou, 730000 China; 2 Key Laboratory for Bio-resource and Eco-environment of Ministry of Education, College of Life Sciences, Sichuan University, Chengdu, 610065 China; 3 Faculty of Veterinary Medicine, Utrecht University, Utrecht, Yalelaan 104, 3584 CM The Netherlands; 4 Department of Statistics, University of Georgia, Athens, GA 30602 USA

## Abstract

The wisent or European bison is the largest European herbivore and is completely cross-fertile with its American relative. However, mtDNA genome of wisent is similar to that of cattle, which suggests that wisent emerged as a hybrid of bison and an extinct cattle-like species. Here, we analyzed nuclear whole-genome sequences of the bovine species, and found only a minor and recent gene flow between wisent and cattle. Furthermore, we identified an appreciable heterogeneity of the nuclear gene tree topologies of the bovine species. The relative frequencies of various topologies, including the mtDNA topology, were consistent with frequencies of incomplete lineage sorting (ILS) as estimated by tree coalescence analysis. This indicates that ILS has occurred and may well account for the anomalous wisent mtDNA phylogeny as the outcome of a rare event. We propose that ILS is a possible explanation of phylogenomic anomalies among closely related species.

## Abstract (web summary)

Kun Wang et al. present a genomic analysis identifying incomplete lineage sorting and hybridization in the mitochondrial DNA of the European bison (wisent). They find that incomplete lineage sorting is the most feasible explanation for the phylogenetic heterogeneity observed in Bovidae.

## Introduction

Mitochondrial DNA (mtDNA) sequences are the most frequently used markers for phylogenetic analysis of animals because of their lack of recombination, high mutation rate and availability of conserved primer sequences^1^. However, mtDNA trees have often been found to be inconsistent with trees inferred from nuclear DNA variation^1–6^. Aside from the potential complications of gene paralogy^7–9^, these discrepancies can be explained by two non-exclusive evolutionary processes, introgressive hybridization and incomplete lineage sorting (ILS)^5,6,10,11^ at the level of individual genes. The first explanation (introgression) is most common for nuclear sister species with divergent mtDNA^4,12^. In this study, we test whether the anomalous mtDNA divergence of the closely related wisent and bison^13^ originated as a result of ancient introgression or ILS.

The wisent (*Bison bonasus*) or European bison is an icon of European wildlife. Since its rescue from extinction in the 1920s, the current population of more than 5000 animals is derived from 12 captive founders^14–16^. Intriguingly, the wisent and American bison are morphologically similar and cross-fertile and have closely related nuclear genes^17,18^, but the mtDNA phylogeny consistently clusters the wisent with the lineage leading to taurine cattle and zebu^19^. Recently, this has been studied in more detail on the basis of ancient DNA and/or whole-genome sequences (WGS)^13^. Wecek et al.^20^ and Gautier et al.^21^ found that a small but variable part of the wisent genome originated from domestic cattle. Another study^12^ suggested that more than 10% of the wisent nuclear genome originated from aurochs, which would support the hybridization hypothesis. However, Massilani et al.^22^ estimated divergence times for these species and concluded that the ILS can account for the mtDNA phylogeny.

In this study, we combine the mitochondrial and nuclear genomic data from previous studies with two new wisent WGS and one new bison WGS and reanalyze the phylogeny. Our analysis started with a detailed discussion of the origin of mtDNA in wisent. We did find recent gene flow, but this did not account for the abnormal phylogeny in mtDNA. We further found a heterogeneous phylogeny across the whole nuclear genome, a small portion of which exhibited a mtDNA-like phylogeny. A coalescent analysis indicated that the phylogeny discordance could be explained well by ILS alone. More generally, our results suggest that the phylogenetic relationship inferred from single genes or whole mtDNA sequences may be misleading, and that the impact of ILS could be evaluated by coalescent analysis when reconstructing the process of speciation.

## Results

### The *Bovini* phylogeny and the anomalous position of the wisent mtDNA genome

**Fig. 1:**
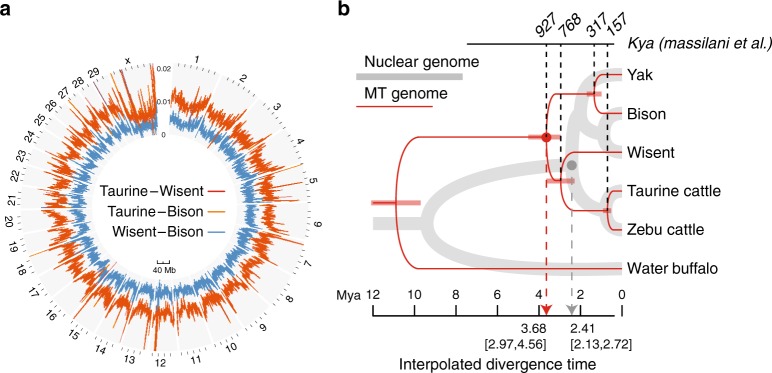
Phylogenetic relationship between wisent and related species. **a** Genetic distances between taurine cattle and wisent, taurine cattle and bison, and wisent and bison, respectively, for a 500 kb sliding window across the genome. The trace for taurine cattle vs wisent lines largely coincides with the trace for taurine cattle vs bison. **b** Maximum likelihood trees of 4,278,251 nuclear fourfold degenerate sites and whole MT genomes with divergence time estimates. The scale indicate in green gives the estimates based on the human mtDNA mutation rate^22^

Genetic distances within a sliding window of 500 kb yielded very similar profiles for the bison–cattle and wisent–cattle comparisons (Pearson's correlation coefficient = 0.91) (Fig. 1a). The genetic distance within the X chromosomal windows was about three-quarters of the autosomal values, which is consistent with the lower effective population size of X chromosomes^23,24^ (Supplementary Table 1). Essentially the same was observed for bison–yak and wisent–yak comparisons (Supplementary Figure 1).

To determine the nuclear phylogenetic history of *Bovini* species, a total of 4,278,251 fourfold degenerate sites in the whole-genome synteny alignment between wisent and five other species in the tribe *Bovini* (water buffalo, taurine cattle, zebu, yak, American bison) were extracted and used to reconstruct a maximum likelihood tree. As in previous studies, the wisent–American bison relationship is supported by a bootstrap percentage of 100% (Fig. 1b). This was confirmed by analysis of separate autosomes (Supplementary Figure 2), species tree estimated by MP-EST(Maximum Pseudo-likelihood for Estimating Species Trees)^25^ and Astral^26^ (Supplementary Figure 3) and by orthologous gene trees (see below).

Using a previously reported mutation rate of 1.1 × 10^−8^ and a generation time of 5 years^27^, we inferred divergence times using a Bayesian-based analysis implemented in BEAST. While the root age was estimated to be about 9 Mya, the divergence time of the taurine cattle and bison lineages was about 2.5 Mya (Fig. 1b). To check the impact of data selection, 10 subsamples of 50,000 fourfold degenerate sites were generated from the original alignment and the estimates of divergence times were consistent across the 10 subsamples (Supplementary Table 2). Although estimated divergence times depend on the computer program, the underlying model, and the calibration (Supplementary Table 2), the estimate for bison and taurine cattle was always ≤3 Mya (Supplementary Table 2).

Using the age of 11 Mya estimated for the *Bovini* based on fossil calibrations^28^, we found that for mtDNA the interpolated divergence time of the taurine–zebu lineage and the bison lineage was about 3.7 Mya (Fig. 1b). Unexpectedly, in a comprehensive tree (Supplementary Figure 4) incorporating 116 mitogenomes of the *Bovini* species, the bison–taurine cattle split was dated at 7.6 Mya. Notably, coalescent times on the basis of human mtDNA mutation rates as the prior^22^ are an order of magnitude lower (Fig. 1b). Estimated population sizes (Supplementary Table 2) were for all species in the range of 135,000 to 750,000.

### Recent introgression between the taurine–zebu cattle and bison–wisent lineages

**Fig. 2:**
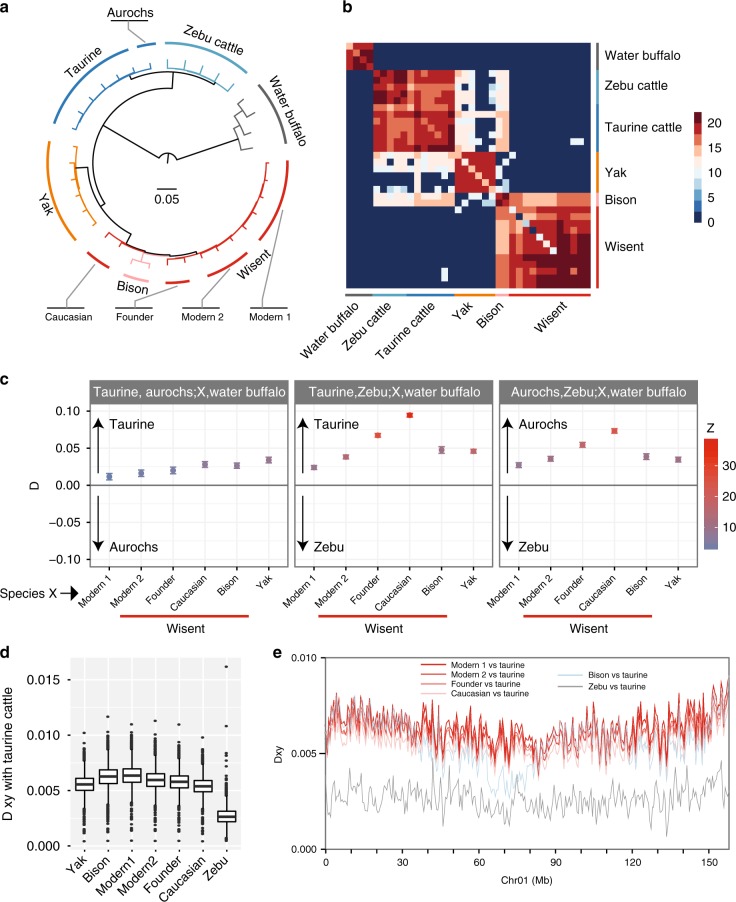
Phylogenetic relationships and inference of gene flow between six species. **a** Tree of allele-sharing distances between 36 individuals based on SNP calling. **b** Log total length of identical-by-descent haplotypes shared by individuals, the dark red corresponding to *e*^20^ = 454 Mbp. **c** ABBA/BABA statistic *D* with the indicated populations. All *D* values shown are significant (*Z*-score > 3, and *p* value < 0.00135). Positive values indicate that species *X* is more related to the first species than to the second species (in the first panel taurine cattle and aurochs, respectively). The respective wisent populations differ in their degree of gene flow with cattle lineages. **d** Pairwise genome-wide *D*_*xy*_ genetic distances between taurine cattle and zebu cattle, yak, American bison, and other wisent populations. *X* indicates the respective species on the *X*-axis. **e** *D*_*xy*_ distances between taurine cattle and zebu, different wisent populations, or American bison along chromosome 1. The Caucasian wisent population has the lowest *D*_*xy*_ in most regions, but not in the regions from 60 to 80 Mb; American bison is remarkably close to taurine cattle, which is not observed in other chromosomes

For a comprehensive analysis of gene flow events, we mapped reads for 36 individuals of the six species, including 31 modern and 5 historic samples (Supplementary Table 3), to the taurine autosomes of the UMD3.1 assembly. After excluding low-quality single-nucleotide polymorphisms (SNPs) and SNPs located in repetitive regions, a total of 94 million bi-allelic SNPs were obtained (Supplementary Table 4). The relationship between these samples (Fig. 2a, Supplementary Figure 5) is consistent with the results in Fig. 1b. However, the Caucasus and founder wisents described by Wecek et al.^20^ differ from modern animals (Fig. 2a). The same topology was obtained using a maximum likelihood approach with Treemix (Supplementary Figure 6), which also indicates gene flow between bison and wisent individuals.

To gain a more detailed insight into the relationships between these species, we searched for identical-by-descent haplotypes using BEAGLE (Fig. 2b). Recently diverged species (taurine and zebu cattle; bison and wisent) exhibit relatively large numbers of identical-by-descent segments. In addition, we found a number of haplotypes shared by the taurine and zebu cattle with bison as well as with yak, which agrees with the presence of taurine and/or zebu mtDNA in their populations^29,30^. In contrast, there were only a few shared identical-by-descent segments between all wisent individuals and cattle.

Analysis of allele frequencies by f3, f4, or ABBA/BABA testing allows greater sensitivity for detecting gene flow between species. Previous studies have inferred gene flow between wisent and taurine cattle, varying from 0.1 to 10%^12,21^. We performed an ABBA/BABA test between these species (Fig. 2c, Supplementary Table 5). We found significant (*Z*-score > 3, and *p* value < 0.00135) gene flows between modern taurine cattle and wisent, bison, or yak with *D* values that are higher than those found for corresponding gene flows to and from aurochs (*Bos primigenius*) or zebu rather than taurine cattle. This suggests that modern taurine cattle are the main source of the recent admixture. The highest value was observed for the Caucasian wisent. On the basis of the f4 ratio test, the proportion of cattle ancestry was estimated to be 2.7%, 2.0%, 4.0%, 1.2%, and 1.0% in yak, bison, Caucasus, wisent founder, and Modern2 populations, respectively (Supplementary Table 6). These trends were confirmed by the normal and outgroup f3 tests (Supplementary Table 7).

A sliding window scan of the genetic distance *D*_*xy*_ between taurine cattle and yak, bison, or wisent populations is shown in Fig. 2d and Supplementary Figure 5. In line with the extent of gene flow, the Caucasian population and Modern1 populations have the lowest and highest *D*_*xy*_ distances to taurine cattle (Fig. 2d). Interestingly, in a part of chromosome 1, bison is closer to taurine cattle (Fig. 2e), which is not observed on other chromosomes (Supplementary Figure 7). This suggests relatively recent gene flow between bison and taurine cattle, which is also consistent with shared identity by descent. These results reveal a complex population history of the wisent with variable levels of cattle ancestry. However, this does not imply that the wisent species emerged as a hybrid species, but rather indicates secondary contacts after the divergence of taurine cattle and zebu or aurochs^21^ or even after the extinction of wisent in the wild^20^.

### Ghost species introgression model of wisent mtDNA origin

**Fig. 3:**
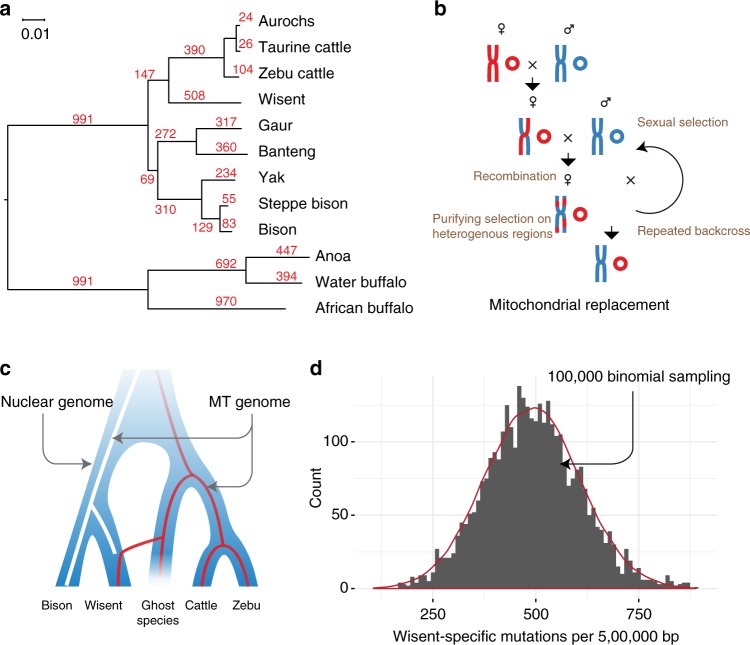
Mitochondrial genome replacement model. **a** Phylogeny of the mtDNA genomes of 12 species and per branch the number of mutations, estimated using RAxML. **b** Hypothetical crossing of two species indicated by red and blue, respectively, proposed to have led to the replacement of mtDNA (small circles). **c** The ghost species introgression model, leading to the transfer of mtDNA from the ghost species to wisent (red line). **d** Histogram of wisent-specific mutations. The red line above the histogram represents the distributions generated by 100,000 binomial sampling based on prior parameters (Methods) and is not expected to fit the observed distribution if the wisent genome contains sequences originating from the ghost species

The recent taurine introgression in wisent varies among different wisent populations, but cannot explain the affinity of the mtDNA from all wisent populations with the lineage leading to taurine and zebu cattle (Figs. 1b and 3a, Supplementary Table 8). However, this does not disprove the possible introgression of a hypothetical ghost species living 6 Mya and carrying an mtDNA that was ancestral to the current wisent mtDNA. We suggest the following scenario (Fig. 3b)^12,19^: male wisent ancestors encounter a herd of a ghost species distantly related to taurine and zebu cattle (Fig. 3a, c) and, because of their large body size, gain the opportunity to mate with the female cows; due to the male sterility of inter-species hybridization in the *Bovini* tribe, only female hybrid offspring reproduce and backcross with the ancestral wisent males; the backcross events continue until the nuclear genome and phenotype of the herd almost completely become wisent-like while the original mtDNA from the ghost species is retained. Purifying selection acting upon highly heterogeneous regions may have accelerated the loss of genes from the ghost species, and so the wisent genome sequences would be expected to have retained a small part of the ghost species genome. However, in the sliding window analysis (Fig. 1a) we did not find more specific mutations in wisent than in bison and the numbers of these mutations in homologous genome segments correlate closely (Fig. 1a, Supplementary Figure 8). In addition, the number of wisent-specific mutations closely follow a binomial distribution (Fig. 3d) which is not compatible with a presumed introgression of a ghost species.

### Phylogenetic discordance of gene trees

**Fig. 4:**
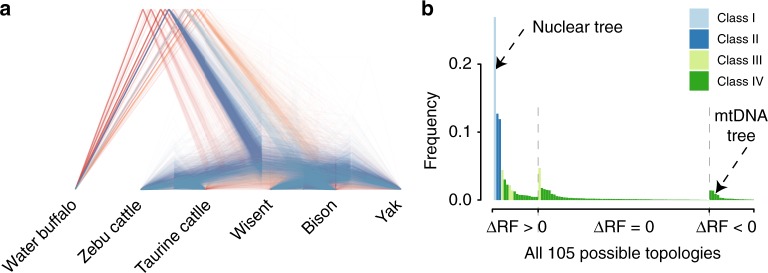
Incomplete lineage sorting model. **a** Gene trees with high bootstrap support. Blue lines indicate the most frequent nuclear phylogeny (Fig. 1b). **b** The empirical frequencies of all 105 possible gene tree topologies ordered according to (1) ΔRF (>0 for gene trees more similar to the nuclear genome tree; 0 for trees equally similar to the nuclear and mtDNA tree; and <0 for trees more similar to the mtDNA tree) and (2) frequency. The four classes of tree topologies are indicated by different colors

To investigate whether the phylogeny of the bovine species is homogeneous across the genome, a total of 15,836 gene trees were constructed for homologous gene sequences extracted from the syntenic alignments. From these gene trees, 3306 have high (>75%) bootstrap support at all nodes. A high level of phylogenetic heterogeneity was found: only 26.9% of all gene trees and 53.5% of the trees with high bootstrap support are consistent with the overall nuclear genome tree (Fig. 4a). There are 105 types of topologies within a rooted binary tree of six terminal branches (Fig. 4b).

We define ΔRF as the difference in the Robinson–Foulds (RF) distance between each topology and the mtDNA tree and the RF distance between each topology and the nuclear genome tree. While most gene trees have a positive ΔRF (71.8%), the proportion of topologies closer to the mtDNA tree (negative ΔRF) was about 6.5%, while the remaining 21.7% of all gene trees had the same RF distance from the nuclear and mtDNA trees (Fig. 4b).

The gene trees were further divided into four classes (Table 1, Supplementary Table 9): Class I, the gene trees overall consistent with the nuclear genome tree, which are not affected by ILS; Class II, two topologies in which ILS joins the taurine–zebu lineage either to the yak or to the bison–wisent lineage; Class III, four topologies in which ILS made yak the sister to the taurine cattle, zebu, bison, and wisent, respectively; and Class IV, two topologies in which wisent joins either the yak or the taurine–zebu lineage (Fig. 4b). We observed a similar number of the two tree topologies in Class II, consistent with ILS^31^. A small but detectable fraction of gene trees (0.87% of all gene trees; 0.39% of the highly supported trees) have the same topology as the mtDNA sequences (wisent linked to the taurine–zebu lineage) (Table 1). The proportion of gene trees that move wisent to the taurine–zebu lineage is similar to the proportion of the trees that move bison to the taurine–zebu lineage, which again is expected by ILS as cause of the phylogenetic discordance across the genome.

**Table 1:** Four classes of observed (empirical) tree topologies with their observed frequencies, observed frequencies among trees with >75% bootstrapping support, and simulated frequencies

Class	Topology	Percent of topology		
		Empirical gene trees	Trees with >75% bootstrap support	Simulated trees
Class I	((((bison,wisent),yak),(taurine,zebu)),buffalo)^a^	26.91%	53.57%	26.54%
Class II	((((taurine,zebu),yak),(bison,wisent)),buffalo)	12.71%	15.46%	14.35%
	((((bison,wisent),(taurine,zebu)),yak),buffalo)	11.90%	15.40%	13.46%
Class III	((((bison,yak),wisent),(taurine,zebu)),buffalo)	4.70%	2.42%	3.48%
	((((wisent,yak),bison),(taurine,zebu)),buffalo)	4.43%	1.60%	3.39%
	((((taurine,yak),zebu),(bison,wisent)),buffalo)	2.17%	1.24%	0.98%
	((((zebu,yak),taurine),(bison,wisent)),buffalo)	1.41%	0.27%	1.01%
Class IV	((((taurine,zebu),wisent),(bison,yak)),buffalo)^b^	0.87%	0.39%	3.10%
	((((taurine,zebu),bison),(wisent,yak)),buffalo)	0.90%	0.45%	3.10%
	Others	33.99%	9.20%	30.61%

^a^Indicates the nuclear genome topology

^b^Indicates the mitochondrial topology

### Multispecies coalescent simulations of ILS

**Fig. 5:**
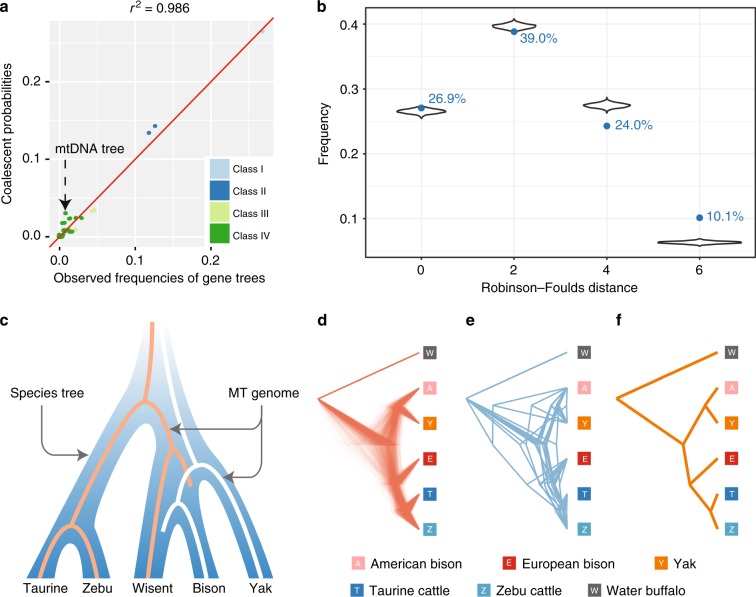
Validation of coalescent simulations. **a** Comparison of simulated frequencies based on coalescent probabilities of gene topologies of different classes with the corresponding observed frequencies. The mtDNA topology is indicated. **b** Comparison of the distributions of Robinson–Foulds distances between the species tree and the observed gene trees (blue numbers and solid blue points) and between the species tree and the gene trees expected on the basis of coalescent simulations (violin plot). The data of violin plot are from 1000 separated simulations (each simulation contains 15,836 gene trees). **c** ILS model showing fixation of different MT genomes in the branches leading to wisent and bison, respectively. **d** Simulated gene trees with the same topology as mtDNA. **e** Observed gene trees with the same topology as the mtDNA tree. **f** Observed mtDNA tree. By scaling all trees in (**d**, **e**, **f**) have the same total length

We used multispecies coalescent simulations to determine the expected gene tree distributions on the basis of a given species tree. A statistical comparison of the distributions generated by the nuclear and mitochondrial species tree (Supplementary Figure 9) with the empirical distribution (Fig. 4b) showed a clearly better fit for the nuclear species tree, which further confirmed the reliability of topology from nuclear genome. For a more detailed comparison of the empirical and simulated distributions from nuclear species tree, we simulated 200,000 gene trees on the basis of the nuclear species tree and compared the resulting frequencies of the topologies against the empirical frequencies (Table 1, Fig. 5a). For the topologies generated by ILS, we found remarkable agreement between the simulated and empirical distributions, although the simulated frequencies of class IV topologies were relatively high. The overall correlation coefficient for the simulated and observed gene trees was 0.98 (Fig. 5a). We also plotted the distribution of the pairwise tree distances between the gene trees and the species tree for both the simulated and observed gene trees and found that the two distance distributions were consistent (Fig. 5b). Therefore, the ILS incorporated in the coalescent model accounts for the genome-wide phylogeny discordance (Fig. 5c) and may very well be promoted by large ancestral population sizes (Supplementary Table 2). Additional support for ILS as an explanation for the mtDNA phylogeny can be derived from the mtDNA topology with branch lengths similar to those in the observed and simulated nuclear genes with the same topology (Fig. 5d–f).

Interestingly, we found that the frequency of mtDNA-like topology was higher in Caucasian and founder wisents (Supplementary Table 10), in agreement with the level of taurine influence (Fig. 2c). In addition, it should be noted that the percentages of mtDNA topology in gene trees may underestimate the level of gene flow in the past, since during subsequent generations the introgressed regions would have been fragmented by recombination events.

## Conclusions

The wisent has had a complex population history. Different populations have variable levels of taurine admixing, which is comparable to the admixture found in American bison. We did not find in the wisent genome any evidence for the 10.6% aurochs DNA as evidence for the ghost species hybrid origin hypothesis^12^. In contrast, the diversity in gene trees as well as the coalescent simulations reveal that ILS accounts for the major part of the genome-wide discordance, while the different levels of gene flow had only a minor influence on this discordance. Our study indicated that ILS should be taken into account when reconstructing the phylogeny of related species.

## Methods

### Data collection

We collected one wisent sample in the form of blood from a male (bbo03) and another wisent sample from the tongue of a female individual (bbo02) at Artis Zoo (Amsterdam) in 2013. We also collected fur of one bison (bbo01) from Yellowstone National Park (USA) in 2006. Genomic DNA was isolated using a Qiagen DNA purification kit. Sequencing libraries with a size of 500 bp were constructed according to the Illumina protocol for each sample. We also collected published data for 33 other bovine samples (Supplementary Table 5). All animal specimens were collected legally. Animal collection and utility protocols were approved by the Animal Ethics Committee of College of Life Science, Lanzhou University, and in accordance with the guidelines from the China Council on Animal Care.

### Phylogeny reconstruction based on synteny alignments and mtDNA

A total of 4.28 Mb fourfold degenerate sites were identified using a custom perl script with the gene annotation files downloaded from the bovine genome database (cattle genome, version UMD3.1). We then extracted these sites for each species in the synteny alignment file stored at Gigadb^32^ (http://gigadb.org/dataset/100254). RAxML^33^ (version 8.2.11, -T 2 -f a -s input -n output -m GTRGAMMAI -x 271828 -N 100 -p 31415 -o water_buffalo) was used for maximum likelihood tree searching and bootstrapping. The same procedure was followed for the separate autosomes. *D*_*xy*_ genetic distances were calculated as the ratio of the number of different bases between two sequences divided by number of all informative sites in a 500 kb window. MtDNA genomes were downloaded from the National Center for Biotechnology Information (NCBI; Supplementary Table 2), aligned with MAFFT^34^ (version 7.205) and the phylogeny was reconstructed using RAxML.

### Divergence time of nuclear genome and mitochondrial genome

Divergence times of the nuclear genome, based on fourfold degenerate sites and whole mtDNA genomes, were first estimated using BEAST^35^ assuming an uncorrelated relaxed clock, running 10,000,000 generations, sampling one generation in every 1000 after discarding the first 25% generations as burn-in. Convergence was checked by Tracer (V1.6, http://tree.bio.ed.ac.uk/software/tracer). We fixed the age of the root to 11 Mya^28^ for both the nuclear and the mtDNA genome. In a separate calculation we used a mutation rate of 1.1 × 10^−9^ per site per generation^22^ and a generation time of 5 years for the nuclear genome.

We also applied IM-CoalHMM^36,37^ to estimate the divergence time and ancestral population size on the basis of fourfold degenerate sites in the nuclear genome under the isolation and isolation with migration models. The same data were analyzed using BPPv4.0^38^, a Bayesian Markov chain Monte Carlo program for estimating species divergence time and species delimitation under the multispecies coalescent model.

### SNP calling and relationship between individuals

The reference sequences were from UMD3.1. We used GEM mapper^39^ (version 20130406-045632) to select the genome locations of 2.3 Gbp of unique sequences. The reads of 36 individuals^20,21,29,32,40–47^ (Supplementary Table 5) were filtered (Scythe, version 0.991, -a Illumina_adapters.fa -q sanger, https://github.com/vsbuffalo/scythe; sickle, version 1.33, pe -t sanger -q 20 -l 50 -n, https://github.com/najoshi/sickle) and aligned to the reference genome with Bwa^48^ (version 0.7.15-r1140, bwa mem -t 32 -R ‘@RG/tID:sampleID/tSM:sampleID/tLB:sampleID’ ref.fa reads1.fq reads2.fq | samtools sort -O bam --threads 16 -o alignment.bam). Duplicated reads were filtered using Picard (version 1.129, default parameters, http://broadinstitute.github.io/picard/). Reads around InDel were realigned by GATK^49^ (version 3.6, default parameters) and the SNPs were genotyped by Samtools^50^ (version 1.3.1). We retained bi-allelic SNPs with a quality larger than 30, 1800 > depth > 72, and missing rate less than 50%.

The distance matrix between samples was generated by Plink^51^ (version 1.90, --cow --distance-matrix) and the neighbor joining (NJ) tree (Fig. 2a) was plotted by Phylip^52^ (version 3.695). Principal component analysis (PCA) (Supplementary Figure 5) was carried out with Plink (version 1.90, --pca --cow) and visualized using the package ggplot2 (version 2.2.1) in R (version 3.2.2).

### Admixture events

TreeMix^53^ (version 1.12) was used to infer the patterns of population splits and historical mixtures with the information on allele frequencies. The tree was rooted with water buffalo and standard errors were estimated using blocks of 500 SNPs. We varied the number of migration events (*m*) between 0 and 5.

We analyzed the D-statistics in the form *D* = (nABBA−nBABA)/(nABBA+nBABA) in a rooted tree ((A, B), C), D) to assess whether population A or B had gene flow with C. If there was no significant gene flow (*Z*-score > 3, and *p* value < 0.00135) between A and C or B and C, the statistic had an expected value of 0. As outgroup D we used the water buffalo. We systematically tried all possible combinations of the wisent or bison samples to test possible migrant events using the qp3Pop program in Admixtools^54^ with default parameters.

We used the program qp3Pop in Admixtools for computing the statistic $f_3\left( {\mathrm {Test};\mathrm {Ref}_1,\mathrm {Ref}_2} \right) = \frac{1}{N}\mathop {\sum}\nolimits_{i = 1}^N {(t_i - r_{1,i})(t_i - r_{2,i})}$, where *N* is the number of SNPs, *t*_*i*_, *r*_1,*i*_ and *r*_2,*i*_ are the allele frequencies for the *i*^th^ SNP in the populations Test, Ref_1_, and Ref_2_, respectively, to determine whether there was evidence that the Test population was derived from an admixture of populations related to Ref_1_ and Ref_2_. A significant negative statistic (*Z*-score < −3, and *p* value < 0.00135) provides unambiguous evidence of gene flow between the Test populations. We assessed the significance of the *f*_3_ statistics via the *Z*-score using a block jackknife and a block size of 5 Mb. We also applied an outgroup-*F*_3_ test *F*_3_(*O*;*A*,*X*), where *O* is an outgroup population of *A* and *X*, and a higher *F*_3_ score indicates more shared alleles. We tried different wisent and bison populations as *X* and cattle as *A* in order to determine which wisent population has the more ancient mixture with the cattle lineage.

We used the qpF4ratio program from Admixtools for computing the *f*_4_ ratio test in order to estimate the mixing proportions of an admixture event. For five populations with a phylogeny relationship with (((*A*, *B*), *C*), *O*) and *X* is supposed to be the mixed population of *B* and *C*, the admixture proportion *α* (proportion from population *B*) could be inferred from *α* = *f*_4_(*A*, *O*; *X*, *C*)/*f*_4_(*A*, *O*; *B*, *C*).

### Species-specific mutation

The species-specific mutations were extracted from synteny alignment files and counted in a 500 kb sliding window; 1769 out of 3530 windows with less than 250 kb informative sites were removed to simulate the distributions of the number of wisent-specific mutations per window. In the absence of introgression, the probability of mutation can be assumed to follow a normal distribution with a mean value of 494 and standard variation of 117, derived from the prior wisent-specific mutation distribution. A total of 100,000 binomial simulations were performed and all data were scaled to a fragment length of 500 kb.

### Construction of gene trees

The gene sequences were extracted from the synteny alignment file and the phylogeny trees were reconstructed using RAxML. A total of 17,729 of genes were extracted, 15,836 of which had a length greater than or equal to 300 bp for all species; these were used to reconstructed the gene trees. To plot all gene trees together, we first converted all of them into ultra-metric trees, separately, using the Phybase package^55^. We used Densitree^56^ to plot these trees together (Fig. 4a).

### Species tree estimation

The species tree was reconstructed from the collection of the maximum likelihood gene trees using the coalescent program MP-EST v2.0^25^. To evaluate the uncertainty of species tree estimation, 100 bootstrap samples were generated from bootstrap gene trees built by RAxML. The bootstrap samples were then used as the input data to build MP-EST. The MP-ESTs were summarized in a majority rule consensus tree, in which the bootstrap support was calculated for each internal branch. We also applied ASTRAL^26^ v5.5.9 with default parameters to infer the species tree.

### The likelihood ratio test for discrepancy between the nuclear and mtDNA trees

We used the likelihood ratio test to show that the discrepancy between the nuclear and mtDNA trees was significant (*Z*-score > 3, and *p* value < 0.00135) and the sequence data strongly favored the nuclear tree. Goodness of fit for the nuclear tree (or the mtDNA tree) was evaluated by comparing the distribution of the maximum likelihood gene trees with the distribution of the gene trees simulated from the nuclear tree (or the mtDNA tree). Specifically, branch lengths in coalescent units were fitted to the nuclear and mtDNA trees, respectively, using MP-EST. Given the two species trees with branch lengths, gene trees were simulated under the multispecies coalescent model using the function sim.coaltree.sp in the R phylogenetic package Phybase. We conducted a likelihood ratio test to evaluate the fit of the two simulated distributions to the empirical distribution of gene trees (Supplementary Figure 9a). Since the number of trees with six taxa is 105, let *X* = {*x*_1_,…,*x*_105_}, in which *x*_*i*_ is the frequency of tree *i* in the distribution of maximum likelihood gene trees. Similarly, let *P*_*j*_ = {*p*_*j*1_,…,*p*_*j*105_} and *j* = {mtDNA,nuclear}, in which *p*_*ji*_ is the probability of tree *i* in the distribution of gene trees simulated for the mtDNA tree (*j* = mtDNA) or the nuclear tree (*j* = nuclear). Given the probabilities *P*_*j*_, the observed frequencies *X* have a multinomial distribution. In the likelihood ratio test, the null hypothesis was represented by the mtDNA tree (*τ*_*m*_) and the alternative hypothesis by the nuclear tree (*τ*_*n*_). The test statistic is *t* = 2(*L*(*τ*_*n*_) − *L*(*τ*_*m*_)), in which *L*(*τ*_*m*_) and *L*(*τ*_*n*_) are the log-likelihoods of the mtDNA and nuclear trees. Since the test involves tree topologies, the asymptotic null distribution of the test statistic is not a χ^2^ distribution. To approximate the null distribution, we generated 1000 parametric bootstrap samples by simulating gene trees from the multinomial distribution with the probabilities *P*_mtDNA_ expected from the mtDNA tree. For each bootstrap sample, we calculated the test statistic t^*^ and the *p* value was equal to the proportion of t^*^ > *t*_obs_, in which *t*_obs_ is the test statistic calculated from the observed frequencies *X* (Supplementary Figure 9b).

### Goodness of fit of the multispecies coalescent model

We compared the empirical distribution of gene trees with the distribution of gene trees expected from the multispecies coalescent model. If the multispecies coalescent model was a good fit to the empirical gene trees, the gene trees expected from the multispecies coalescent model would be consistent with the empirical gene trees. Let $\hat P = \left( {\widehat {p_1}, \ldots ,\widehat {p_{105}}} \right)$ be the frequencies of empirical gene trees. Let *P* = (*p*_1_,…,*p*_105_) be the probabilities of gene trees expected from the multispecies coalescent model. We calculated the correlation between the observed frequencies $\hat P$ and the expected probabilities *P* of gene trees. We fitted a linear function of the form $\hat P = cP$ in R. The multispecies coalescent model is a good fit to the empirical gene trees, if the coefficient *c* is close to 1. Moreover, we calculated the pairwise tree distances between the empirical gene trees and the species tree. The observed distances were then compared with the expected distances between the coalescent gene trees and the species tree (Fig. 5b).

### Choice of outgroup

In order to test whether the choice of outgroup (water buffalo) influences the results, we aligned the genome of goat, *Capra hircus* (ENSEMBL v92), to the cattle genome and performed the same phylogeny analysis for each as above. About 10.3% of the gene trees exhibited an anomalous position of water buffalo (Supplementary Figure 10) and the distribution pattern of the other gene trees was not affected. Moreover, the frequencies of observed gene trees were also consistent with the frequency of simulated gene trees under the ILS model if goat was used as the outgroup. This justifies the choice of water buffalo as the outgroup.

### Code availability

The custom scripts for ILS simulation have been deposited in https://github.com/wk8910/ILS_simulation.

## Electronic supplementary material

Supplementary files

## Data Availability

The sequence data have been deposited in the NCBI SRA database with accession numbers SRR3530515, SRR3531976, and SRR3532327.
