# Supplementary material for: Incomplete lineage sorting rather than hybridization explains the inconsistent phylogeny of the wisent
Source: Commun Biol. 2018 Oct 19;1:169. doi: 10.1038/s42003-018-0176-6 (PMC6195592; doi:10.1038/s42003-018-0176-6)
Supplement: Supplementary file 1 — Supplementary files [file 42003_2018_176_MOESM1_ESM.pdf]

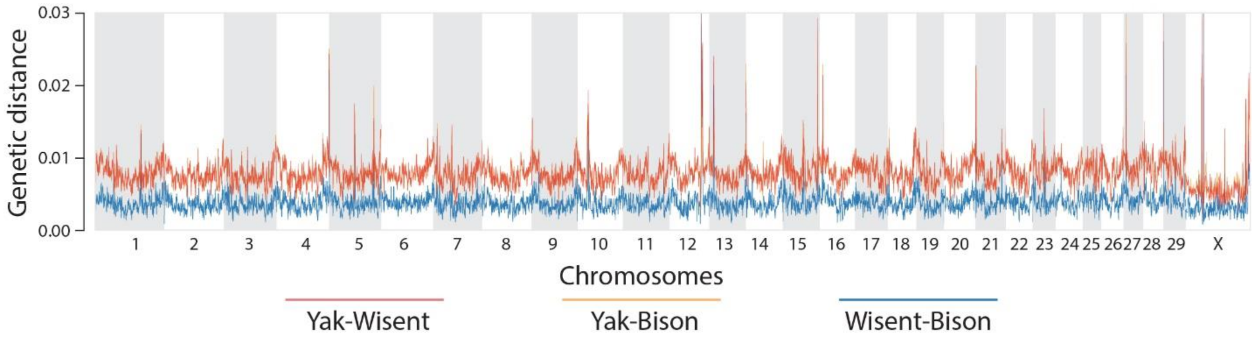

Supplementary Figure 1 | Genetic distances between the yak and wisent, yak and bison, and wisent and bison 500kb sliding window across the genome.

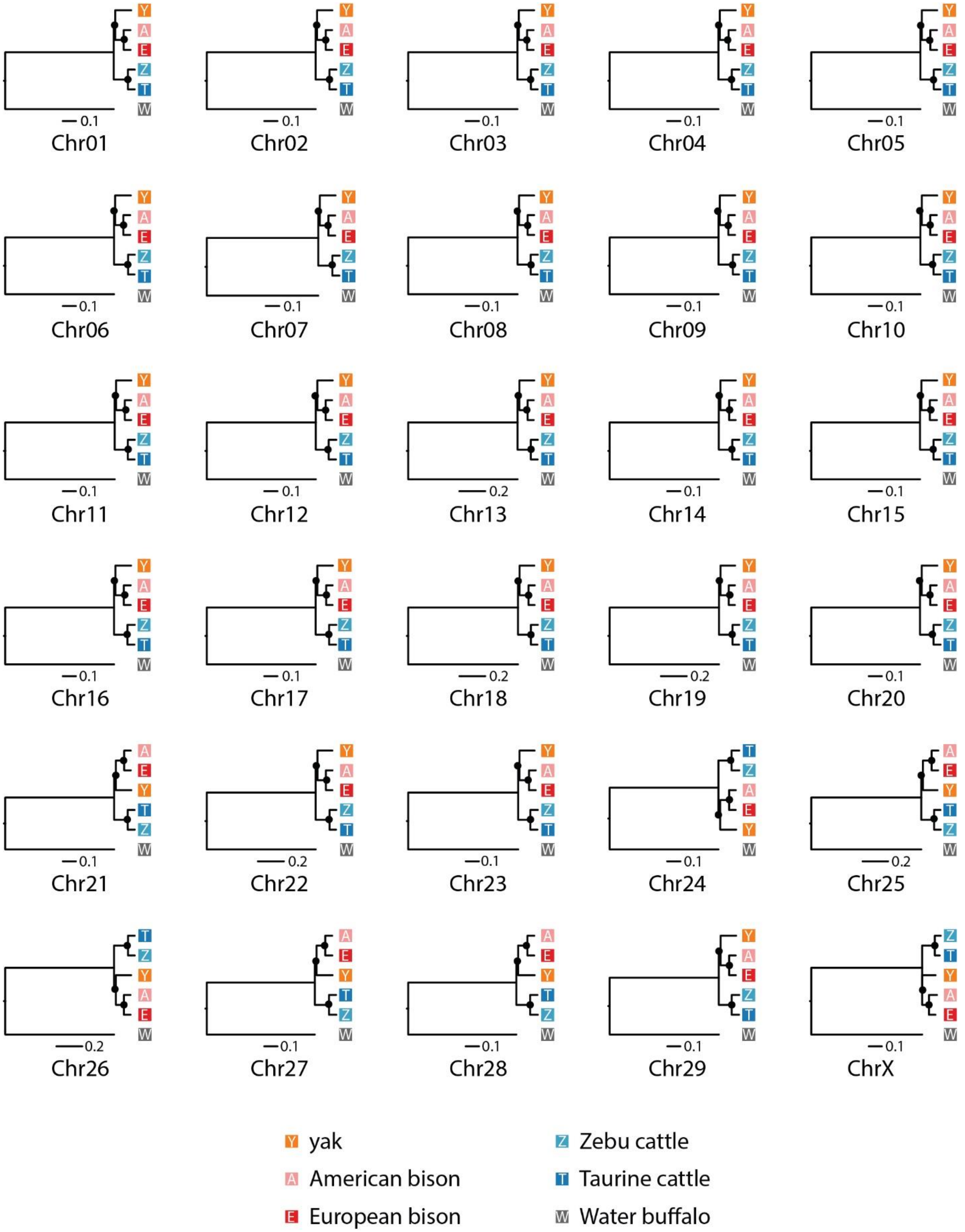

Supplementary Figure 2 | ML trees from synteny alignment data for separate autosomes.

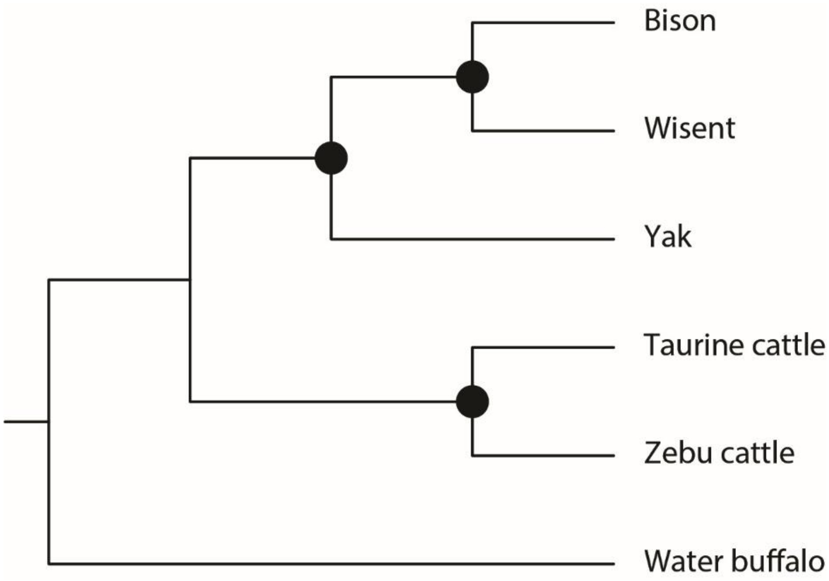

Supplementary Figure 3 | The species tree reconstructed with ASTRAL with the default parameters. The black solid dot in the node indicate a 100% of posterior probability.

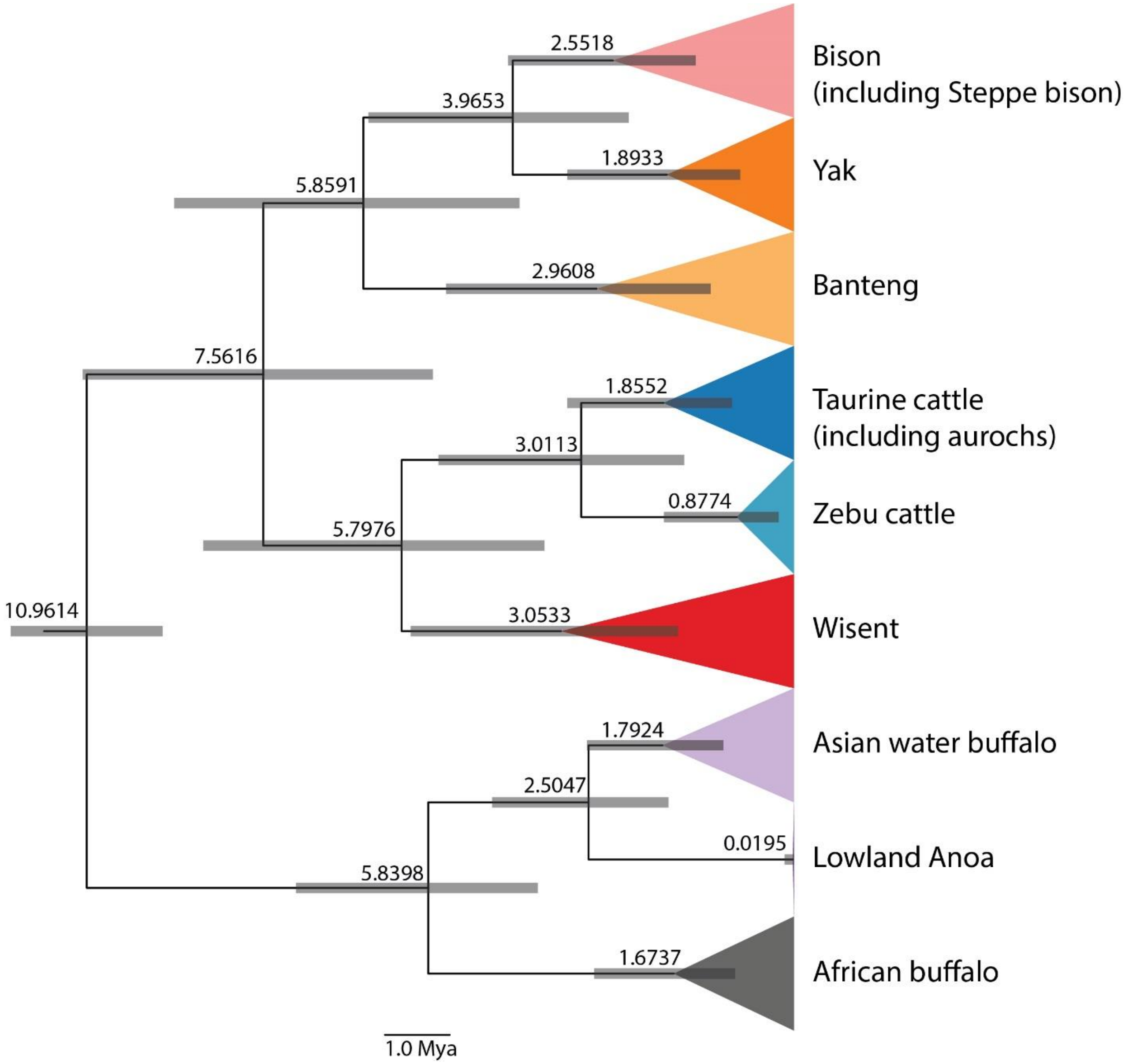

Supplementary Figure 4 | Tree topologies and divergence time estimated from 116 mtDNA sequences estimated with BEAST. Grey bars indicate the uncertainty (95% CI). The sequences included: "Bison (*Bison bison*): KX451358.1, KX451367.1, GU946991.1, GU946979.1, GU946985.1, GU946988.1, GU946984.1, GU946986.1, JN632601.1, GU946980.1, GU946992.1, GU946997.1, KX451353.1, KX451352.1, KX451362.1, KX451359.1, KX451355.1, GU946978.1, GU946976.1, NC_012346.1; Steppe bison (*Bison priscus*): KX269136.1, KX269122.1, KX269128.1, KR350472.1, NC_027233.1, KX269126.1, KX269145.1, KX269119.1, KX269131.1, KX269134.1, KX898020.1, KX269117.1, KX898018.1, KX269137.1, KX269116.1, KX269123.1, KX269113.1, KX898010.1, KX269121.1, KX898014.1, KX592174.1, KX269130.1, KX269109.1, KX269141.1; Yak (*Bos grunniens*): GQ464255.1, GQ464312.1, GQ464247.1, GQ464314.1, GQ464286.1, GQ464256.1, KM658599.1, KM233416.1, GQ464292.1, GQ464267.1, GQ464295.1, KX232522.1, GQ464282.1, GQ464293.1, GQ464300.1, KR052524.1; Wisent (*Bison bonasus*): KX898006.1, KX592184.1, KX553934.1, KX592177.1, JN632602.1, KX898015.1, KX898011.1, KX592188.1, KX592175.1; Yak: GQ464255.1, GQ464312.1, GQ464247.1, GQ464314.1, GQ464286.1, GQ464256.1, KM658599.1, KM233416.1, GQ464292.1, GQ464267.1, GQ464295.1, KX232522.1, GQ464282.1, GQ464293.1, GQ464300.1, KR052524.1; Zebu: AF492350.1, GU256940.1, JN817304.1; Banteng (*Bos javanicus*): JN632606.1, FJ997262.1, AB915322.1, NC_012706.1, JN632605.1; Aurochs (*Bos primigenius*): KF525852.1, NC_013996.1, JQ437479.1, GU985279.1; Taurine (*Bos taurus*): DQ124418.1, KT184462.1, KC153972.1, AY676867.1, V00654.1, FJ971088.1, JN817339.1, DQ124415.1, KT184469.1, FJ971082.1, EU177849.1; Asian water buffalo (*Bubalus bubalis*): KX758323.1, KX758393.1, KX758305.1, KX758332.1, KX758402.1, KX758354.1, KX758398.1, KX758357.1, KX758303.1, KX758318.1, KX758356.1, KX758351.1, KX758311.1, KX758350.1, KX758346.1; Lowland Anoa (*Bubalus depressicornis*): EF536351.1, NC_020615.1; African buffalo (*Syncerus caffer*): JQ235527.1, JQ235524.1, JQ235517.1, JQ235547.1, JQ235536.1, JQ235538.1, JQ235509.1".

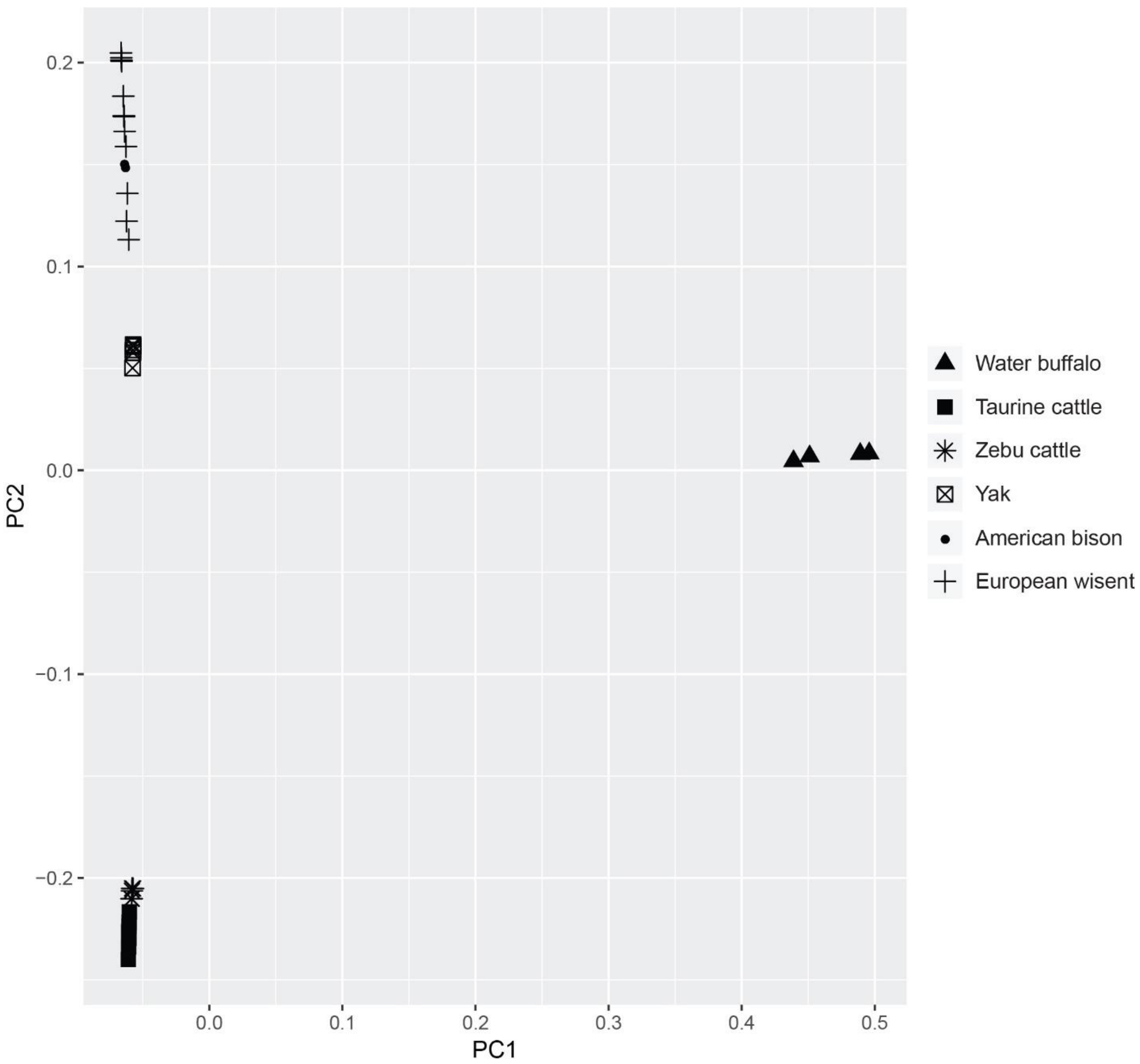

Supplementary Figure 5 | Principal component analysis of 36 bovine genomes.

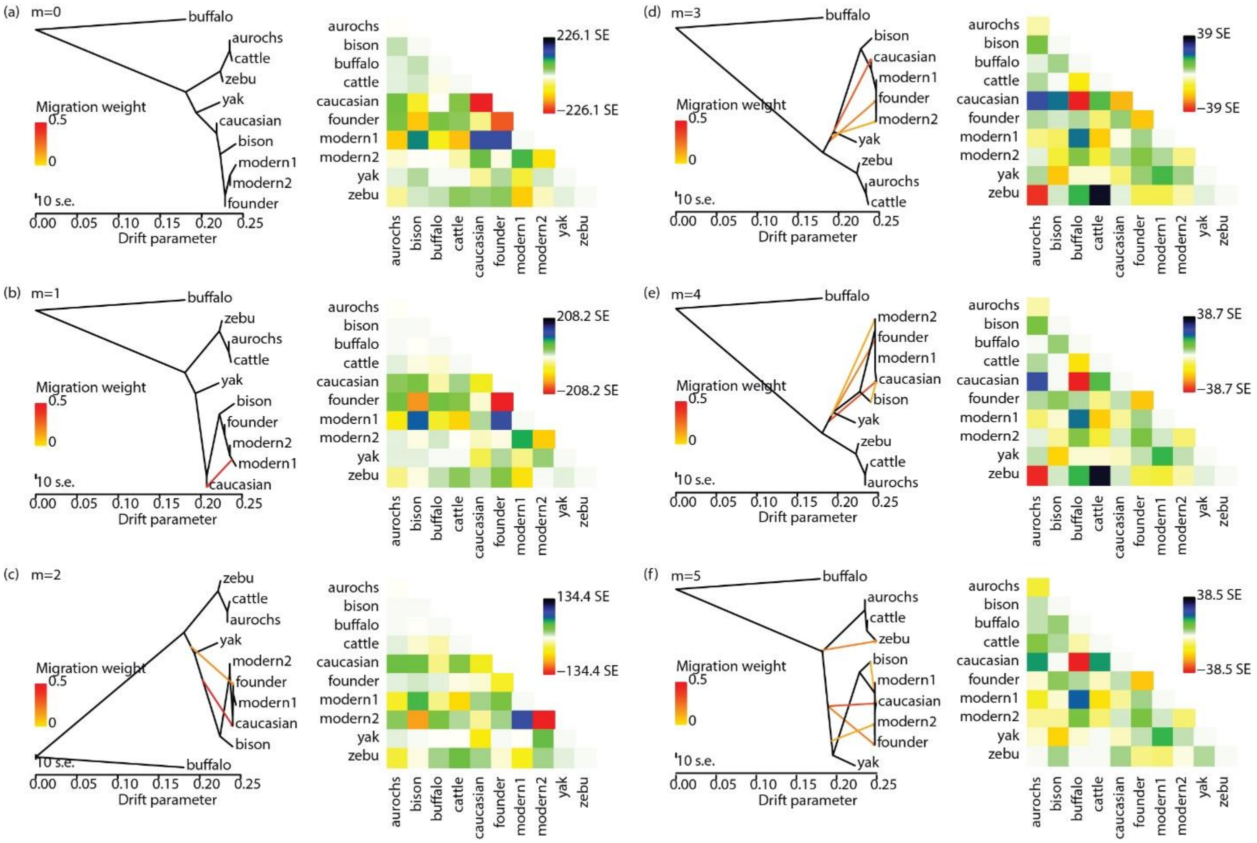

Supplementary Figure 6 | Phylogenetic trees allowing m (0 to 5) gene flow events and corresponding residuals inferred by Treemix.

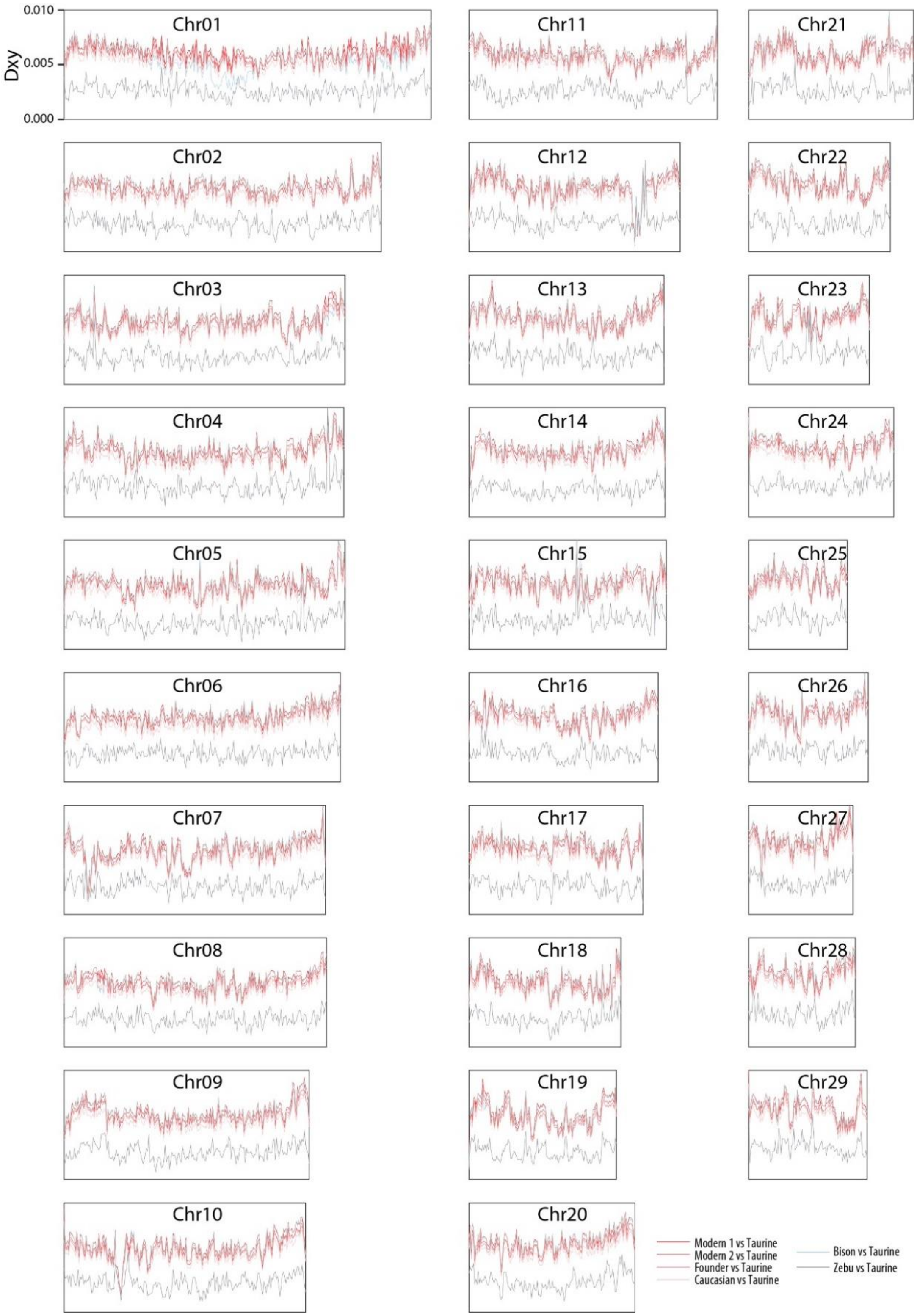

Supplementary Figure 7 | Dxy between taurine cattle and zebu, different wisent populations and bison.

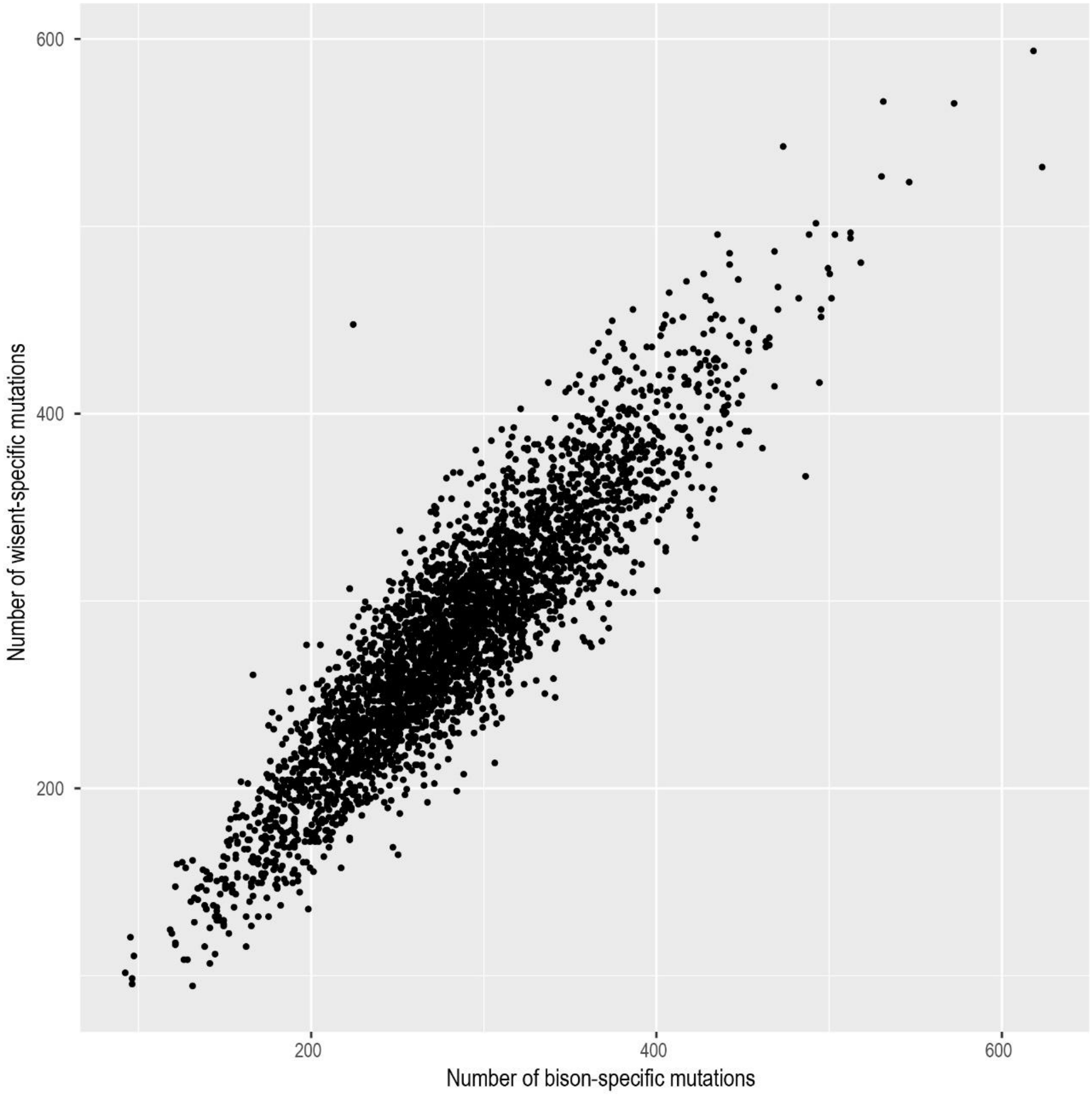

Supplementary Figure 8 | Correlation of the numbers of wisent- and bison-specific mutations within a 500-kb sliding window.

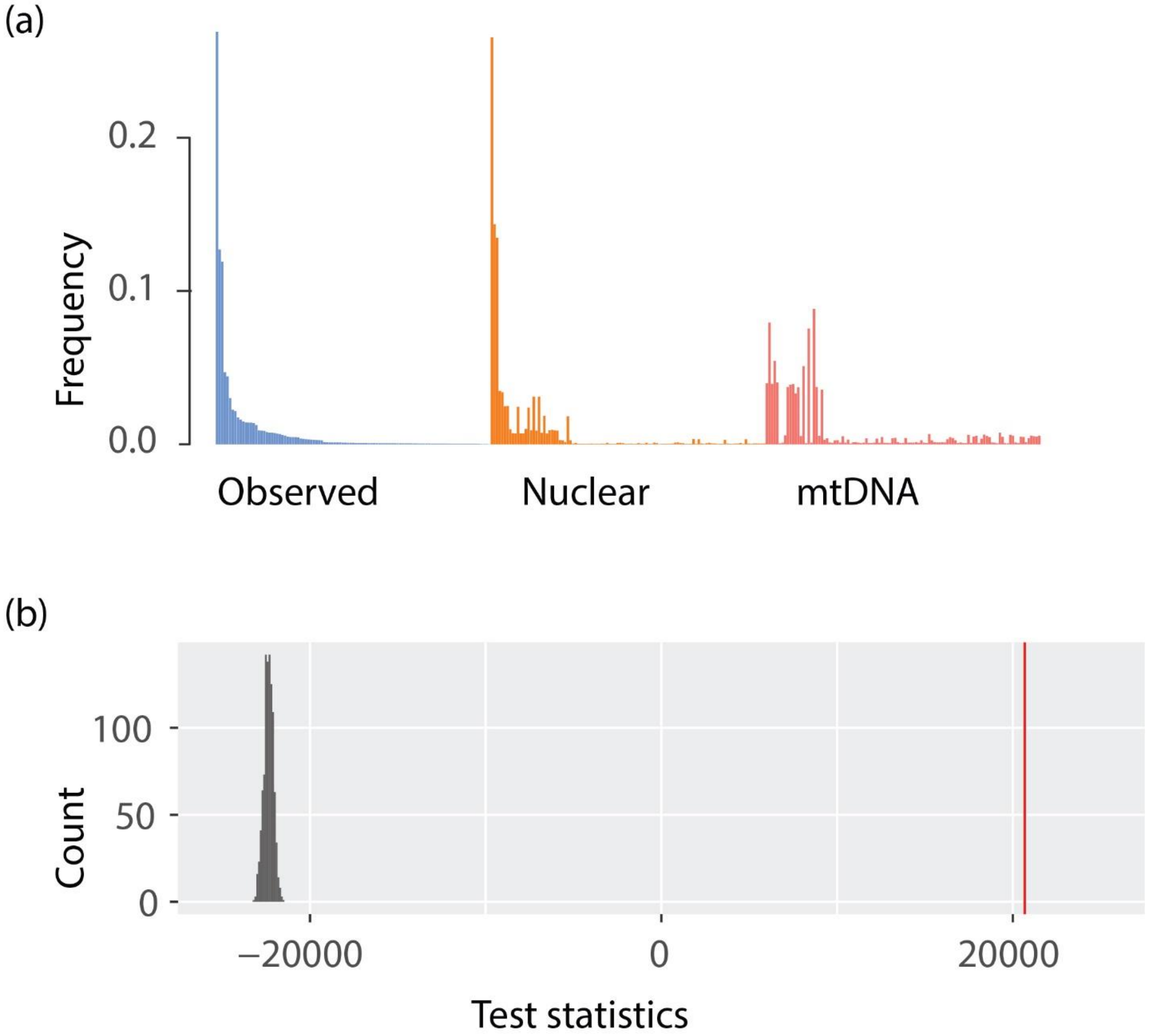

Supplementary Figure 9 | The coalescent analysis of the nuclear genomes of six bovine species. (a) The distributions of the ML gene trees, ofthe gene trees generated on the basis of the nuclear topology, and of the gene trees generated from the mtDNA topologies. This shows that the simulated nuclear tree but not the simulated mtDNA trees fits the ML gene trees. (b) The null distribution of the LRT test statistic for evaluating the fit of the mtDNA species tree to the empirical gene trees; the red line indicates the test statistic value for the observed gene trees.

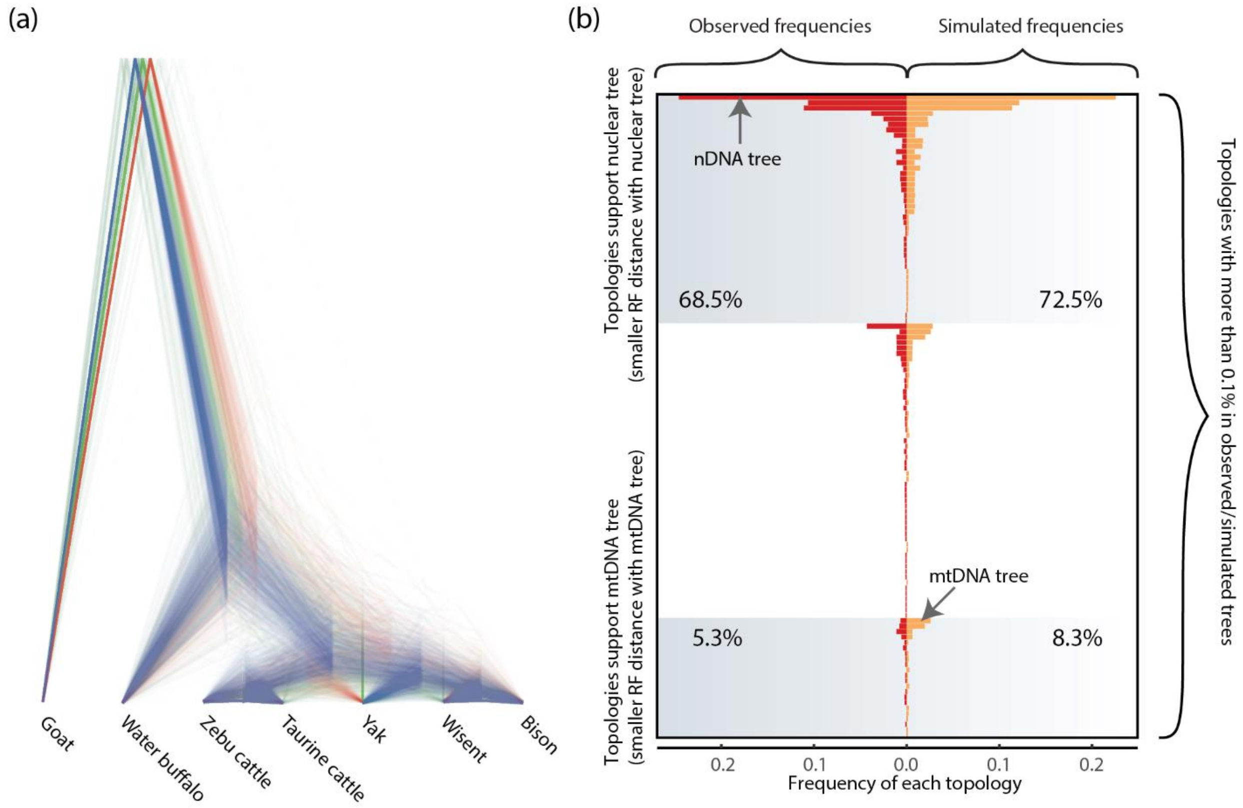

Supplementary Figure 10 | ILS analysis with goat as an outgroup. (a) Gene trees with high bootstrap support. (b) Corresponding frequencies of observed gene trees and simulated gene trees showing essentially the same results as with water buffalo as outgroup (Fig. 4a, b).

Supplementary Table 1 | Genetic distances per autosome between bovine species across the genome.

Chromosome	wisent.bison	wisent.cattle	bison.cattle
chr01	0.00371	0.00941	0.00937
chr02	0.00351	0.00904	0.00901
chr03	0.00355	0.00894	0.00892
chr04	0.00374	0.00936	0.00935
chr05	0.00366	0.00918	0.00914
chr06	0.00392	0.00945	0.00941
chr07	0.00364	0.00916	0.00914
chr08	0.00359	0.00914	0.00911
chr09	0.00373	0.00936	0.00931
chr10	0.00360	0.00897	0.00894
chr11	0.00354	0.00910	0.00910
chr12	0.00385	0.00964	0.00961
chr13	0.00349	0.00927	0.00927
chr14	0.00369	0.00948	0.00945
chr15	0.00394	0.00952	0.00949
chr16	0.00385	0.00945	0.00944
chr17	0.00376	0.00969	0.00968
chr18	0.00352	0.00922	0.00924
chr19	0.00352	0.00927	0.00926
chr20	0.00384	0.00992	0.00990
chr21	0.00379	0.00959	0.00958
chr22	0.00382	0.00964	0.00964
chr23	0.00399	0.00979	0.00977
chr24	0.00386	0.00994	0.00996
chr25	0.00403	0.01009	0.01013
chr26	0.00390	0.00988	0.00988
chr27	0.00431	0.01065	0.01062
chr28	0.00410	0.01054	0.01054
chr29	0.00417	0.01048	0.01043
chrX	0.00315	0.00633	0.00660
autosome	0.00374	0.00944	0.00942

Supplementary Table 2 | Divergence times estimates in Mya by IM-CoalHMM, BPP and BEAST and values based on fossil evidence. BPP and BEAST estimates are averaged over 10 subsamples.

	**Effective population size of common ancestor**		**Divergence time (Mya)**							
	**IMcoalHMM**		**IMcoalHMM**			**BPP with mutation rate**	**BEAST with mutation rate**	**BEAST with fossil data**		**Massilani et al. (2016) using human mtDNA mutation rate**
	**isolation-model**	**isolation by migration-model**	**isolation-model**	**isolation by migration-model**						
**buffalo - wisent**	748,737	602,628	4.44	8.13	**(((bison,wisent),yak), (taurine,zebu)) - buffalo**	7.88 ±0.23	9.60 ±0.16	10.93 ±0.00	**(bison,yak) - (wisent, (taurine,zebu))**	0.927
**buffalo - bison**	747,196	604,526	4.47	8.10						
**buffalo - taurine**	758,837	611,616	4.36	8.04						
**buffalo - yak**	766,371	610,531	4.37	7.60						
**taurine - wisent**	288,524	261,593	1.14	1.74	**((bison,wisent),yak) - (taurine,zebu)**	2.27 ±0.06	2.46 ±0.07	3.42 ±0.30	**wisent - (taurine,zebu)**	0.768
**taurine - bison**	294,717	273,664	1.11	1.54						
**taurine - yak**	307,483	286,224	1.02	1.42						
**zebu - wisent**	271,734	247,608	0.85	1.36						
**zebu - bison**	294,822	289,843	0.73	0.76						
**zebu - yak**	278,329	266,046	0.80	1.16						
**wisent - yak**	293,282	283,312	0.71	0.93	**(bison,wisent) - yak**	1.72 ±0.07	2.06 ±0.09	2.64 ±0.17	**(bison,wisent) - yak**	0.317
**bison - yak**	291,381	284,451	0.74	0.88						
**bison - wisent**	185,690	185,579	0.09	0.10	**bison - wisent**	0.71 ±0.05	0.92 ±0.05	1.19 ±0.10	**bison - wisent**	0.246
**taurine - zebu**	139,120	134,265	0.02	0.06	**taurine - zebu**	0.66 ±0.07	0.80 ±0.07	1.09 ±0.20	**taurine - zebu**	0.157

Supplementary Table 3 | Information on individuals used for SNP calling.

sample name	Species	group	mean depth	coverage	SNP calling rate	Heterozygosity	accession ID	Source
bbi01	Bison	bison	11.84	97.84%	98.74%	0.12%	SRR3530515	**newly sequenced**
bbi02	Bison	bison	8.75	97.34%	99.01%	0.14%	SRR4035288	Kalbfleisch and Heaton 2013
bbu01	Water buffalo	buffalo	10.83	94.03%	96.21%	0.45%	SRR034232	Tantia, et al. 2011
buffalo001	Water buffalo	buffalo	10.17	93.81%	96.49%	0.41%	SRR4477879	Whitacre, et al. 2017
buffalo002	Water buffalo	buffalo	11.98	95.31%	97.89%	0.26%	SRR4477891	Whitacre, et al. 2017
buffalo003	Water buffalo	buffalo	13.35	95.52%	97.98%	0.22%	SRR4477896	Whitacre, et al. 2017
bos_pri	Taurine cattle	aurochs	7.38	96.16%	93.01%	0.10%	SRR2463292	Park, et al. 2015
bta01	Taurine cattle	cattle	11.39	96.98%	98.46%	0.06%	DRR000429	Tsuda, et al. 2013
bta02	Taurine cattle	cattle	5.93	90.44%	90.39%	0.10%	ERR489556	Noyes, et al. 2015
bta03	Taurine cattle	cattle	6.00	95.13%	96.37%	0.11%	SRR1262533	Brøndum, et al. 2014
bta04	Taurine cattle	cattle	5.33	95.76%	97.21%	0.10%	SRR1262621	Brøndum, et al. 2014
bta05	Taurine cattle	cattle	6.74	96.59%	98.77%	0.10%	SRR1262795	Brøndum, et al. 2014
bta06	Taurine cattle	cattle	12.37	96.84%	99.60%	0.11%	SRR934411	Lee, et al. 2014
bbo006	Wisent	caucasian	2.09	82.81%	79.97%	0.08%	SRR4996860	Wecek, et al. 2017
bbo7	Wisent	caucasian	3.45	78.05%	78.33%	0.11%	SRR4996862	Wecek, et al. 2017
bbo4	Wisent	founder	5.98	90.02%	91.21%	0.13%	SRR5003743	Wecek, et al. 2017
bbo5	Wisent	founder	3.86	80.25%	80.21%	0.10%	SRR5003913	Wecek, et al. 2017
bbo01	Wisent	modern1	19.92	97.94%	99.21%	0.08%	SRR3530566	Wang, et al. 2017
bbo02	Wisent	modern1	12.25	96.03%	98.99%	0.09%	SRR3531976	**newly sequenced**
bbo03	Wisent	modern1	5.81	92.22%	94.70%	0.11%	SRR3532327	**newly sequenced**
bbo04	Wisent	modern1	8.46	94.24%	98.93%	0.07%	SRR3178073	Gautier, et al. 2016
bbo05	Wisent	modern1	9.69	94.51%	99.08%	0.08%	SRR3178074	Gautier, et al. 2016
bbo1	Wisent	modern2	4.07	92.74%	95.06%	0.09%	SRR4853874	Gautier, et al. 2016
bbo2	Wisent	modern2	4.03	93.20%	95.49%	0.10%	SRR4861203	Gautier, et al. 2016
bbo3	Wisent	modern2	3.64	91.93%	93.91%	0.10%	SRR4861351	Gautier, et al. 2016
bgr01	Yak	yak	4.08	92.92%	94.51%	0.13%	SRX1054970	Qiu, et al. 2015
bgr02	Yak	yak	2.93	89.85%	88.38%	0.12%	SRX1055971	Qiu, et al. 2015
bgr03	Yak	yak	4.28	94.22%	95.47%	0.13%	SRX1056023	Qiu, et al. 2015
bgr04	Yak	yak	4.09	93.38%	94.49%	0.13%	SRX1056032	Qiu, et al. 2015
bgr05	Yak	yak	4.26	93.32%	94.25%	0.13%	SRX1056033	Qiu, et al. 2015
bgr06	Yak	yak	4.03	92.95%	94.10%	0.13%	SRX1056034	Qiu, et al. 2015
bin02	Zebu cattle	zebu	9.95	97.05%	99.84%	0.14%	SRR3524756	Kim, et al. 2017
zebu001	Zebu cattle	zebu	10.49	98.33%	99.80%	0.23%	SRR3524810	Kim, et al. 2017
zebu002	Zebu cattle	zebu	10.48	97.21%	99.91%	0.39%	SRR3546787	Kim, et al. 2017
zebu003	Zebu cattle	zebu	10.35	98.32%	99.79%	0.25%	SRR3592092	Kim, et al. 2017
zebu004	Zebu cattle	zebu	10.41	98.38%	99.84%	0.25%	SRR3694658	Kim, et al. 2017

Supplementary Table 4 | Summary statistics of SNPs.

Type	Number
intergenic_region	55,219,037
intron_variant	30,892,278
upstream_gene_variant	3,622,238
downstream_gene_variant	2,876,499
5_prime_UTR_variant	221,465
3_prime_UTR_variant	604,901
synonymous_variant	469,778
missense_variant	265,519
stop_gained	4,146
stop_lost	1,298
start_lost	714
splice_region_variant&intron_variant	59,614
intragenic_variant	15,836
5_prime_UTR_premature_start_codon_gain_variant	10,653
splice_region_variant&synonymous_variant	9,549
missense_variant&splice_region_variant	4,769
splice_region_variant	3,865
splice_donor_variant&intron_variant	1,225
stop_retained_variant	901
splice_acceptor_variant&intron_variant	871
stop_gained&splice_region_variant	87
stop_lost&splice_region_variant	78
initiator_codon_variant&non_canonical_start_codon	78
splice_region_variant&stop_retained_variant	60
splice_acceptor_variant&splice_donor_variant&intron_variant	38
start_lost&splice_region_variant	14
splice_region_variant&initiator_codon_variant&non_canonical_start_codon	2

Supplementary Table 5 | D-statistic tests. An absolute value of Z-value greater than 3 means significant signal of gene flow.

A	B	C	O	D (>0 A close to C; <0 B close to X)	Z-value
bison	yak	cattle	buffalo	-0.014	-1.600
bison	yak	zebu	buffalo	-0.016	-2.278
bison	yak	aurochs	buffalo	-0.012	-1.431
modern1	yak	cattle	buffalo	-0.074	-22.348
modern1	yak	zebu	buffalo	-0.062	-20.739
modern1	yak	aurochs	buffalo	-0.063	-18.574
modern2	yak	cattle	buffalo	-0.042	-12.256
modern2	yak	zebu	buffalo	-0.037	-12.082
modern2	yak	aurochs	buffalo	-0.036	-10.210
founder	yak	cattle	buffalo	-0.014	-4.378
founder	yak	zebu	buffalo	-0.024	-8.127
founder	yak	aurochs	buffalo	-0.017	-4.952
caucasian	yak	cattle	buffalo	0.067	19.773
caucasian	yak	zebu	buffalo	0.046	14.896
caucasian	yak	aurochs	buffalo	0.064	18.940
cattle	aurochs	modern1	buffalo	0.011	3.127
cattle	aurochs	modern2	buffalo	0.015	4.478
cattle	aurochs	founder	buffalo	0.019	5.294
cattle	aurochs	caucasian	buffalo	0.027	9.488
cattle	aurochs	bison	buffalo	0.027	9.960
cattle	aurochs	yak	buffalo	0.035	11.979
cattle	zebu	modern1	buffalo	0.022	11.283
cattle	zebu	modern2	buffalo	0.036	19.114
cattle	zebu	founder	buffalo	0.065	36.457
cattle	zebu	caucasian	buffalo	0.093	54.128
cattle	zebu	bison	buffalo	0.046	11.510
cattle	zebu	yak	buffalo	0.045	22.358
aurochs	zebu	modern1	buffalo	0.026	9.668
aurochs	zebu	modern2	buffalo	0.034	13.138
aurochs	zebu	founder	buffalo	0.053	20.720
aurochs	zebu	caucasian	buffalo	0.072	32.304
aurochs	zebu	bison	buffalo	0.037	10.938
aurochs	zebu	yak	buffalo	0.033	13.285
modern1	bison	cattle	buffalo	-0.119	-7.185
modern1	bison	zebu	buffalo	-0.094	-7.387
modern1	bison	aurochs	buffalo	-0.105	-6.401
modern2	bison	cattle	buffalo	-0.052	-3.030
modern2	bison	zebu	buffalo	-0.040	-3.077
modern2	bison	aurochs	buffalo	-0.045	-2.665
founder	bison	cattle	buffalo	-0.008	-0.536
founder	bison	zebu	buffalo	-0.021	-1.854
founder	bison	aurochs	buffalo	-0.015	-1.011
caucasian	bison	cattle	buffalo	0.122	7.186
caucasian	bison	zebu	buffalo	0.092	7.229
caucasian	bison	aurochs	buffalo	0.117	7.102
modern1	modern2	cattle	buffalo	-0.151	-38.013
modern1	founder	cattle	buffalo	-0.154	-47.758
modern1	caucasian	cattle	buffalo	-0.298	-96.771
modern2	founder	cattle	buffalo	-0.058	-21.635
modern2	caucasian	cattle	buffalo	-0.227	-80.064

founder	caucasian	cattle	buffalo	-0.162	-62.337
modern1	modern2	zebu	buffalo	-0.116	-38.817
modern1	founder	zebu	buffalo	-0.092	-33.076
modern1	caucasian	zebu	buffalo	-0.226	-80.706
modern2	founder	zebu	buffalo	-0.018	-7.263
modern2	caucasian	zebu	buffalo	-0.169	-64.360
founder	caucasian	zebu	buffalo	-0.137	-56.204
modern1	modern2	aurochs	buffalo	-0.137	-35.085
modern1	founder	aurochs	buffalo	-0.124	-38.024
modern1	caucasian	aurochs	buffalo	-0.279	-87.186
modern2	founder	aurochs	buffalo	-0.037	-12.579
modern2	caucasian	aurochs	buffalo	-0.213	-70.336
founder	caucasian	aurochs	buffalo	-0.158	-56.866

Supplementary Table 6 | f4 ratio tests. X is admixed from B and C, alpha is the percent of X come from B (Patterson et al. 2012). The phylogeny follows (((A,B),C),O).

A	X	B	C	O	alpha	Z-value
zebu	modern2	cattle	modern1	buffalo	0.010975	51.435
zebu	founder	cattle	modern1	buffalo	0.012966	38.84
zebu	caucasian	cattle	modern1	buffalo	0.040149	85.514
zebu	bison	cattle	modern1	buffalo	0.019707	13.283
zebu	yak	cattle	modern1	buffalo	0.027767	23.416

Supplementary Table 7 | The result of F3-test.

Source 1	Source 2	Target	f 3	Z
**Outgroup f3-test (Outgroup F3 test was used to test the extent of shared genes between Source1 and Source2; a higher value indicate more shared genes between Source1 and Source2)**				
cattle	modern1	buffalo	3.741575	120.747
cattle	yak	buffalo	3.757806	117.915
cattle	modern2	buffalo	3.760409	121.885
cattle	bison	buffalo	3.766019	118.673
cattle	founder	buffalo	3.819809	123.79
cattle	caucasian	buffalo	3.916396	121.262
**f3-test (Normal F3 test was used to test if the target was admixed from Source1 and Source2; a significant negative value indicate the Target was admixed)**				
cattle	modern1	modern2	-0.20636	-90.624
cattle	modern1	founder	-0.25139	-132.697
cattle	modern1	caucasian	-0.25521	-195.844
cattle	modern1	bison	0.352324	21.872
cattle	modern1	yak	0.661094	86.23

Supplementary Table 8 | Mitochondrial sequences used in this study.

Latin name	Common name	accession ID	Source
Bos primigenius	Aurochs	GU985279.1	Edwards, et al. 2010
Bos taurus	Taurine cattle	AB074968.1	Mannen, et al. 2003
Bos indicus	Zebu cattle	AY126697.1	Canavez, et al. 2012
Bison bonasus	Wisent	NC_014044.1	Zeyland, et al. 2012
Bos gaurus	Gaur	JN632604.1	Hassanin, et al. 2012
Bos javanicus	Banteng	JN632606.1	Hassanin, et al. 2012
Bos grunniens	Yak	KR011113.1	Guo, et al. 2016
Bison priscus	Steppe bison	KR350472.1	Kirillova, et al. 2015
Bison bison	Bison	GU947006.1	Douglas, et al. 2011
Bubalus depressicornis	Anoa	EF536351.1	Hassanin, et al. 2012
Bubalus bubalis	Water buffalo	AY488491.1	Parma, et al. 2004
Syncerus caffer	African buffalo	JQ235521.1	Heller, et al. 2012

Supplementary Table 9 | Topologies of gene trees.

Topology	Empirical gene trees		Trees with >75% bootstrap support		Simulated trees				
**Topology**	**Count**	**Percent**	**Count**	**Percent**	**Count**	**Percent**	**Class**	**RF distance close to ntGenome**	**RF distance close to mtGenome**
((((bison,wisent),yak),(taurine,zebu)),buffalo)	4262	26.91%	1771	53.57%	53079	26.54%	Class I	0	4
((((taurine,zebu),yak),(bison,wisent)),buffalo)	2013	12.71%	511	15.46%	28699	14.35%	Class II	2	4
((((bison,wisent),(taurine,zebu)),yak),buffalo)	1885	11.90%	509	15.40%	26924	13.46%	Class II	2	4
((((bison,yak),wisent),(taurine,zebu)),buffalo)	744	4.70%	80	2.42%	6952	3.48%	Class III	2	2
((((wisent,yak),bison),(taurine,zebu)),buffalo)	701	4.43%	53	1.60%	6771	3.39%	Class III	2	4
((((taurine,yak),zebu),(bison,wisent)),buffalo)	344	2.17%	41	1.24%	1950	0.98%	Class III	4	6
((((zebu,yak),taurine),(bison,wisent)),buffalo)	223	1.41%	9	0.27%	2011	1.01%	Class III	4	6
**((((taurine,zebu),wisent),(bison,yak)),buffalo)**	**138**	**0.87%**	**13**	**0.39%**	**6194**	**3.10%**	**Class IV**	**4**	**0**
((((taurine,zebu),bison),(wisent,yak)),buffalo)	143	0.90%	15	0.45%	6199	3.10%	Class IV	4	4
(((((bison,wisent),yak),zebu),taurine),buffalo)	477	3.01%	64	1.94%	4962	2.48%	Class IV	2	6
(((((bison,wisent),yak),taurine),zebu),buffalo)	356	2.25%	62	1.88%	4991	2.50%	Class IV	2	6
((((taurine,zebu),(wisent,yak)),bison),buffalo)	235	1.48%	12	0.36%	4877	2.44%	Class IV	4	4
((((bison,yak),(taurine,zebu)),wisent),buffalo)	217	1.37%	7	0.21%	4790	2.40%	Class IV	4	2
(((((taurine,zebu),yak),wisent),bison),buffalo)	278	1.76%	25	0.76%	1440	0.72%	Class IV	4	4
((((bison,wisent),zebu),(taurine,yak)),buffalo)	119	0.75%	9	0.27%	3733	1.87%	Class IV	4	6
(((((taurine,zebu),yak),bison),wisent),buffalo)	254	1.60%	16	0.48%	1427	0.71%	Class IV	4	4
((((bison,wisent),taurine),(zebu,yak)),buffalo)	73	0.46%	11	0.33%	3646	1.82%	Class IV	4	6
(((((bison,wisent),taurine),zebu),yak),buffalo)	199	1.26%	10	0.30%	1831	0.92%	Class IV	4	6
(((((taurine,zebu),wisent),bison),yak),buffalo)	223	1.41%	9	0.27%	1424	0.71%	Class IV	4	2
(((((taurine,zebu),bison),wisent),yak),buffalo)	226	1.43%	7	0.21%	1433	0.72%	Class IV	4	4
(((((bison,wisent),zebu),taurine),yak),buffalo)	140	0.88%	9	0.27%	1775	0.89%	Class IV	4	6
((((bison,wisent),(taurine,yak)),zebu),buffalo)	115	0.73%	5	0.15%	1847	0.92%	Class IV	4	6
(((((bison,wisent),zebu),yak),taurine),buffalo)	105	0.66%	6	0.18%	1807	0.90%	Class IV	4	6
(((((bison,wisent),taurine),yak),zebu),buffalo)	96	0.61%	8	0.24%	1774	0.89%	Class IV	4	6
(((((taurine,zebu),wisent),yak),bison),buffalo)	118	0.75%	9	0.27%	1415	0.71%	Class IV	4	2

((((bison,wisent),(zebu,yak)),taurine),buffalo)	110	0.69%	3	0.09%	1871	0.94%	Class IV	4	6
(((((taurine,zebu),bison),yak),wisent),buffalo)	126	0.80%	5	0.15%	1476	0.74%	Class IV	4	4
(((((bison,yak),wisent),zebu),taurine),buffalo)	88	0.56%	3	0.09%	542	0.27%	Class IV	4	4
(((((wisent,yak),bison),zebu),taurine),buffalo)	73	0.46%	4	0.12%	518	0.26%	Class IV	4	6
(((((wisent,yak),bison),taurine),zebu),buffalo)	74	0.47%	3	0.09%	479	0.24%	Class IV	4	6
(((((bison,yak),wisent),taurine),zebu),buffalo)	80	0.51%	0	0.00%	506	0.25%	Class IV	4	4
(((((wisent,yak),zebu),taurine),bison),buffalo)	59	0.37%	0	0.00%	322	0.16%	Class IV	6	6
((((bison,zebu),(taurine,wisent)),yak),buffalo)	70	0.44%	0	0.00%	118	0.06%	Class IV	6	6
((((bison,yak),zebu),(taurine,wisent)),buffalo)	13	0.08%	1	0.03%	660	0.33%	Class IV	6	4
((((wisent,yak),zebu),(bison,taurine)),buffalo)	12	0.08%	0	0.00%	636	0.32%	Class IV	6	6
((((wisent,yak),taurine),(bison,zebu)),buffalo)	6	0.04%	1	0.03%	643	0.32%	Class IV	6	6
((((bison,yak),(taurine,wisent)),zebu),buffalo)	30	0.19%	1	0.03%	338	0.17%	Class IV	6	4
((((bison,zebu),(wisent,yak)),taurine),buffalo)	22	0.14%	2	0.06%	364	0.18%	Class IV	6	6
(((((bison,taurine),zebu),wisent),yak),buffalo)	55	0.35%	0	0.00%	66	0.03%	Class IV	6	6
(((((taurine,yak),zebu),bison),wisent),buffalo)	50	0.32%	1	0.03%	60	0.03%	Class IV	6	6
((((bison,yak),(zebu,wisent)),taurine),buffalo)	32	0.20%	0	0.00%	339	0.17%	Class IV	6	4
((((bison,taurine),(wisent,yak)),zebu),buffalo)	31	0.20%	0	0.00%	336	0.17%	Class IV	6	6
(((((zebu,wisent),taurine),bison),yak),buffalo)	51	0.32%	0	0.00%	70	0.04%	Class IV	6	4
(((((taurine,yak),zebu),wisent),bison),buffalo)	42	0.27%	2	0.06%	61	0.03%	Class IV	6	6
(((((taurine,wisent),zebu),bison),yak),buffalo)	47	0.30%	1	0.03%	55	0.03%	Class IV	6	4
((((bison,zebu),taurine),(wisent,yak)),buffalo)	17	0.11%	1	0.03%	431	0.22%	Class IV	6	6
((((bison,yak),taurine),(zebu,wisent)),buffalo)	8	0.05%	0	0.00%	599	0.30%	Class IV	6	4
((((taurine,wisent),(zebu,yak)),bison),buffalo)	44	0.28%	0	0.00%	131	0.07%	Class IV	6	6
(((((taurine,yak),wisent),zebu),bison),buffalo)	48	0.30%	0	0.00%	56	0.03%	Class IV	6	6
(((((bison,yak),taurine),zebu),wisent),buffalo)	29	0.18%	0	0.00%	275	0.14%	Class IV	6	4
((((taurine,wisent),zebu),(bison,yak)),buffalo)	21	0.13%	0	0.00%	375	0.19%	Class IV	6	2
(((((bison,yak),zebu),taurine),wisent),buffalo)	20	0.13%	1	0.03%	286	0.14%	Class IV	6	4
(((((wisent,yak),taurine),zebu),bison),buffalo)	25	0.16%	0	0.00%	283	0.14%	Class IV	6	6
((((bison,taurine),zebu),(wisent,yak)),buffalo)	17	0.11%	0	0.00%	370	0.19%	Class IV	6	6
(((((taurine,wisent),bison),zebu),yak),buffalo)	40	0.25%	0	0.00%	65	0.03%	Class IV	6	6
((((bison,taurine),(zebu,wisent)),yak),buffalo)	36	0.23%	0	0.00%	115	0.06%	Class IV	6	6

(((((bison,zebu),taurine),wisent),yak),buffalo)	39	0.25%	0	0.00%	52	0.03%	Class IV	6	6
(((((zebu,yak),taurine),bison),wisent),buffalo)	32	0.20%	1	0.03%	62	0.03%	Class IV	6	6
((((bison,zebu),(taurine,yak)),wisent),buffalo)	26	0.16%	1	0.03%	121	0.06%	Class IV	6	6
((((zebu,wisent),taurine),(bison,yak)),buffalo)	10	0.06%	0	0.00%	366	0.18%	Class IV	6	2
(((((bison,yak),zebu),wisent),taurine),buffalo)	17	0.11%	0	0.00%	276	0.14%	Class IV	6	4
(((((bison,zebu),wisent),taurine),yak),buffalo)	32	0.20%	0	0.00%	76	0.04%	Class IV	6	6
(((((taurine,wisent),zebu),yak),bison),buffalo)	33	0.21%	0	0.00%	63	0.03%	Class IV	6	4
(((((wisent,yak),zebu),bison),taurine),buffalo)	17	0.11%	0	0.00%	263	0.13%	Class IV	6	6
(((((zebu,yak),taurine),wisent),bison),buffalo)	27	0.17%	1	0.03%	68	0.03%	Class IV	6	6
(((((bison,zebu),taurine),yak),wisent),buffalo)	32	0.20%	0	0.00%	59	0.03%	Class IV	6	6
(((((taurine,wisent),bison),yak),zebu),buffalo)	29	0.18%	0	0.00%	64	0.03%	Class IV	6	6
((((bison,taurine),(zebu,yak)),wisent),buffalo)	25	0.16%	0	0.00%	97	0.05%	Class IV	6	6
(((((wisent,yak),taurine),bison),zebu),buffalo)	7	0.04%	0	0.00%	322	0.16%	Class IV	6	6
(((((bison,yak),taurine),wisent),zebu),buffalo)	11	0.07%	0	0.00%	269	0.13%	Class IV	6	4
(((((bison,taurine),wisent),yak),zebu),buffalo)	26	0.16%	0	0.00%	69	0.03%	Class IV	6	6
(((((bison,taurine),yak),zebu),wisent),buffalo)	22	0.14%	1	0.03%	55	0.03%	Class IV	6	6
(((((bison,taurine),wisent),zebu),yak),buffalo)	26	0.16%	0	0.00%	59	0.03%	Class IV	6	6
((((taurine,yak),(zebu,wisent)),bison),buffalo)	18	0.11%	0	0.00%	120	0.06%	Class IV	6	6
(((((taurine,yak),wisent),bison),zebu),buffalo)	23	0.15%	0	0.00%	51	0.03%	Class IV	6	6
(((((bison,zebu),wisent),yak),taurine),buffalo)	22	0.14%	0	0.00%	56	0.03%	Class IV	6	6
((((bison,taurine),wisent),(zebu,yak)),buffalo)	8	0.05%	1	0.03%	171	0.09%	Class IV	6	6
(((((taurine,yak),bison),wisent),zebu),buffalo)	16	0.10%	1	0.03%	64	0.03%	Class IV	6	6
((((taurine,wisent),bison),(zebu,yak)),buffalo)	12	0.08%	0	0.00%	174	0.09%	Class IV	6	6
(((((zebu,wisent),bison),yak),taurine),buffalo)	19	0.12%	0	0.00%	68	0.03%	Class IV	6	6
(((((taurine,wisent),yak),bison),zebu),buffalo)	20	0.13%	0	0.00%	54	0.03%	Class IV	6	6
((((bison,zebu),wisent),(taurine,yak)),buffalo)	10	0.06%	0	0.00%	180	0.09%	Class IV	6	6
(((((zebu,wisent),bison),taurine),yak),buffalo)	20	0.13%	0	0.00%	52	0.03%	Class IV	6	6
((((bison,zebu),yak),(taurine,wisent)),buffalo)	10	0.06%	0	0.00%	178	0.09%	Class IV	6	6
(((((zebu,yak),wisent),taurine),bison),buffalo)	19	0.12%	0	0.00%	64	0.03%	Class IV	6	6
(((((bison,taurine),zebu),yak),wisent),buffalo)	20	0.13%	0	0.00%	42	0.02%	Class IV	6	6
(((((zebu,wisent),taurine),yak),bison),buffalo)	17	0.11%	0	0.00%	55	0.03%	Class IV	6	4

(((((zebu,yak),wisent),bison),taurine),buffalo)	16	0.10%	0	0.00%	59	0.03%	Class IV	6	6
((((bison,taurine),yak),(zebu,wisent)),buffalo)	5	0.03%	0	0.00%	195	0.10%	Class IV	6	6
((((zebu,yak),wisent),(bison,taurine)),buffalo)	7	0.04%	0	0.00%	167	0.08%	Class IV	6	6
((((zebu,wisent),yak),(bison,taurine)),buffalo)	6	0.04%	0	0.00%	167	0.08%	Class IV	6	6
((((zebu,wisent),bison),(taurine,yak)),buffalo)	7	0.04%	0	0.00%	151	0.08%	Class IV	6	6
((((taurine,yak),bison),(zebu,wisent)),buffalo)	3	0.02%	0	0.00%	182	0.09%	Class IV	6	6
(((((zebu,yak),bison),wisent),taurine),buffalo)	13	0.08%	0	0.00%	55	0.03%	Class IV	6	6
((((taurine,wisent),yak),(bison,zebu)),buffalo)	3	0.02%	0	0.00%	167	0.08%	Class IV	6	6
(((((taurine,wisent),yak),zebu),bison),buffalo)	12	0.08%	0	0.00%	51	0.03%	Class IV	6	6
((((zebu,yak),bison),(taurine,wisent)),buffalo)	2	0.01%	0	0.00%	172	0.09%	Class IV	6	6
(((((taurine,yak),bison),zebu),wisent),buffalo)	10	0.06%	0	0.00%	59	0.03%	Class IV	6	6
(((((zebu,yak),bison),taurine),wisent),buffalo)	9	0.06%	0	0.00%	61	0.03%	Class IV	6	6
(((((bison,zebu),yak),taurine),wisent),buffalo)	9	0.06%	0	0.00%	58	0.03%	Class IV	6	6
((((taurine,yak),wisent),(bison,zebu)),buffalo)	0	0.00%	0	0.00%	162	0.08%	Class IV	6	6
(((((bison,taurine),yak),wisent),zebu),buffalo)	8	0.05%	0	0.00%	60	0.03%	Class IV	6	6
(((((zebu,wisent),yak),bison),taurine),buffalo)	8	0.05%	0	0.00%	56	0.03%	Class IV	6	6
(((((zebu,wisent),yak),taurine),bison),buffalo)	7	0.04%	0	0.00%	62	0.03%	Class IV	6	6
(((((bison,zebu),yak),wisent),taurine),buffalo)	6	0.04%	0	0.00%	49	0.02%	Class IV	6	6

Supplementary Table 10 | The distribution of tree topologies inferred using different wisent individuals. The “*” means Spearman's correlation co-efficient between the distribution of topologies of the population in the first column and modern1 population.

Gene trees from	same as nuclear like tree	same as mt-like tree	more congruent to mt-like tree	r2 with modern1 (bbo01)*
modern1(bbo04)	26.66%	0.72%	5.82%	0.997
modern2(bbo1)	26.78%	0.75%	5.79%	0.997
founder(bbo4)	26.20%	0.82%	6.01%	0.996
founder(bbo5)	25.59%	1.00%	6.64%	0.996
caucasian(bbo006)	25.93%	0.88%	6.52%	0.996
caucasian(bbo7)	23.54%	2.13%	10.79%	0.992
Simulated gene trees	26.91%	3.10%	8.92%	0.986
